# Mass Spectrometry Imaging Combined With Single‐Cell Transcriptional Profiling Reveals the Multidimensional Spatial Distributions and Biosynthetic Pathways of Medicinal Components in *Andrographis paniculata*

**DOI:** 10.1111/pbi.70534

**Published:** 2026-01-08

**Authors:** HaiSheng Zeng, MeiHui Shi, ZhiRong Chen, XueJing Sun, HuiJie Zhang, Yue Huang, YuCheng Chen, Jun Ren, HuiLing Huang, Almaz Borjigidai, Man Zhang, SuJuan Duan, Yi‐Jun Chen, Hong‐Lei Jin, Hong‐Bin Wang

**Affiliations:** ^1^ School of Pharmaceutical Sciences/State Key Laboratory of Traditional Chinese Medicine Syndrome Guangzhou University of Chinese Medicine Guangzhou China; ^2^ Institute of Medical Plant Physiology and Ecology/Key Laboratory of Chinese Medicinal Resource From Lingnan, School of Pharmaceutical Sciences Guangzhou University of Chinese Medicine Guangzhou China; ^3^ National Immunological Laboratory for Traditional Chinese Medicine Affiliated Hospital of Youjiang Medical University for Nationalities Baise China; ^4^ Chinese Medicine Guangdong Laboratory Guangdong Hengqin Zhuhai China

**Keywords:** *Andrographis paniculata* ( *A. paniculata* ), andrographolide, desorption electrospray ionisation mass spectrometry imaging (DESI‐MSI), metabolomics, single‐cell RNA sequencing

## Abstract

The synthesis and accumulation of active ingredients in medicinal plants are distributed in specific organs, tissues, and cell types, which are important for the exploitation of medicinal plants. However, the fine distribution of active ingredients is difficult to know. Here, the system of mass spectrometry imaging (MSI) integrated with single‐cell RNA sequencing was established for the first time in *Andrographis paniculata* ( *A. paniculata* ), a medicinal plant widely utilised in China and Southeast Asia. MSI shows specific distribution of andrographolides in *A. paniculata* , with higher accumulation in non‐veinal leaf regions and outer stem cortex (leaf > stem; outer > inner cortex), as validated by LC‐QQQ‐MS/MS assays. Leaf scRNA‐seq demonstrates that *ApCPS2* (the key terpene synthase for andrographolide biosynthesis) exhibits pronounced cell‐type‐specific expression in photosynthetic mesophyll subclusters, indicating mesophyll cells as the primary site for light‐modulated andrographolide production. Interestingly, light may enhance the accumulation of andrographolide biosynthesis, confirming the light sensitivity of metabolism in mesophyll cells. This study explores medicinal components' multidimensional spatial distributions and biosynthetic pathways in *A. paniculata* via MSI combined with single‐cell technology, providing a novel strategy for determining plant metabolites' fine synthesis and distribution.

## 1 Introduction

The spatial and temporal heterogeneity in the synthesis and accumulation of plant secondary metabolites across diverse organs, tissues, and cell types is a well‐documented phenomenon, reflecting functional specialisation and ecological adaptation (Parrish et al. 2022; Zhou et al. 2022). As it was reported, peltate glandular trichomes in *Mentha* spp. served as biosynthetic hubs for volatile terpenoids and phenolic derivatives (Liu et al. 2023), while the stems of *Catharanthus roseus* facilitate terpenoid indole alkaloid (TIA) production through a multicellular assembly line involving epidermal cells, idioblasts, and laticifers (Yamamoto et al. 2016). Recent spatial transcriptomic studies in *Taxus* leaves further demonstrated the predominant expression of taxol biosynthetic genes in palisade mesophyll cells (Zhan et al. 2023). Collectively, these findings highlight the integral role of cellular compartmentalization and intercellular transport in shaping the profile, quantity, and distribution of secondary metabolites (Guirimand et al. 2011; Noman et al. 2021; Qiu et al. 2018; Wu et al. 2021). However, a comprehensive understanding of the in situ spatial architecture of metabolic pathways remains technically challenging.

Advances in mass spectrometry imaging (MSI) have revolutionised spatial metabolomics by enabling label‐free, untargeted visualisation of metabolite distributions within biological tissues at high spatial resolution (Chen et al. 2024; Unsihuay et al. 2021). Techniques such as matrix‐assisted laser desorption/ionisation (MALDI)‐MSI and desorption electrospray ionisation (DESI)‐MSI have been employed to delineate the tissue‐specific localization of key metabolites, thereby providing critical insights into biosynthetic regulation and physiological adaptation (Bhandari et al. 2015; Dreisbach et al. 2021; Garrett et al. 2016; Montini et al. 2020; Righetti et al. 2022). These approaches have successfully mapped the accumulation patterns of terpenoids, alkaloids, and other specialised metabolites across various plant organs (e.g., roots, leaves, flowers), revealing complex spatiotemporal dynamics in metabolite production and storage (Conceição et al. 2021; Liao et al. 2019; Wu et al. 2022). However, conventional MSI methodologies are often constrained by limitations in spatial resolution and sensitivity, particularly for detecting low‐abundance metabolites at subcellular levels, thus impeding detailed mechanistic studies on metabolic trafficking and regulation (Guo et al. 2023; Yin et al. 2019; Zhou et al. 2021).

The advent of single‐cell RNA sequencing (scRNA‐seq) has addressed these challenges by providing unprecedented resolution in profiling gene expression and metabolic activities at the cellular level (Zhang et al. 2020). scRNA‐seq allows for the dissection of cellular heterogeneity, identification of rare cell types, and reconstruction of transcriptional networks driving metabolic diversification (Deshmukh et al. 2021; Liang et al. 2023; Liew et al. 2024; Sun et al. 2022; Wang et al. 2020). Integration of scRNA‐seq with spatial transcriptomics and metabolomics has further enhanced our ability to correlate genetic regulation with metabolic output across tissue contexts (Chen et al. 2025; Zhao et al. 2024). In particular, this integrated approach is increasingly applied in medicinal plants to elucidate the biosynthetic pathways of high‐value natural products (Li et al. 2023; Wang, Zhang, et al. 2025; Zhan et al. 2023).

*Andrographis paniculata (A. paniculata)*, a perennial herbaceous species in the Acanthaceae family, is a widely used medicinal plant in clinical practice that is extensively distributed across China, Southeast Asia, and South Asia (Jadhav and Karuppayil 2021; Jiang et al. 2021; Kumar et al. 2021). Its bioactive constituents, predominantly diterpenoid lactones such as andrographolide, neoandrographolide, and deoxyandrographolide, exhibit anti‐inflammatory, antiviral, and anticancer activities (Zhang et al. 2021). These compounds are mainly synthesised and accumulated in the leaves and stems (Yu et al. 2024), yet their precise spatial distribution patterns and the regulatory mechanisms underlying their biosynthesis remain inadequately characterised. Previous studies have identified several key enzymes and transcription factors involved in the andrographolide pathway, including genes encoding diterpene synthases and cytochrome P450 monooxygenases (Lv et al. 2025; Wang, Liang, et al. 2025), but cell‐type‐specific expression and metabolic flux dynamics are still poorly understood.

So, in this study, we established the system of MSI and single‐cell methodologies to systematically investigate the spatial distribution of andrographolide‐type components across distinct tissues and their regulatory mechanisms underlying biosynthesis and accumulation in *A. paniculata* . We first applied DESI‐MSI to map the tissue‐specific accumulation of these metabolites in leaf and stem sections. We then performed scRNA‐seq to identify distinct cell populations and characterise their transcriptomic profiles, thereby reconstructing putative regulatory circuits involved in andrographolide synthesis. Finally, we conducted light intensity intervention experiments to assess environmental influences on metabolic accumulation. Our work not only provides a methodological framework for studying spatial metabolism in non‐model plants but also offers valuable insights into the bioengineering of diterpenoid production and the cultivation of *A. paniculata* under optimised environmental conditions.

## 2 Results

### 2.1 The Content of Andrographolide Is Higher in Leaf and Stem Epidermis

**FIGURE 1:**
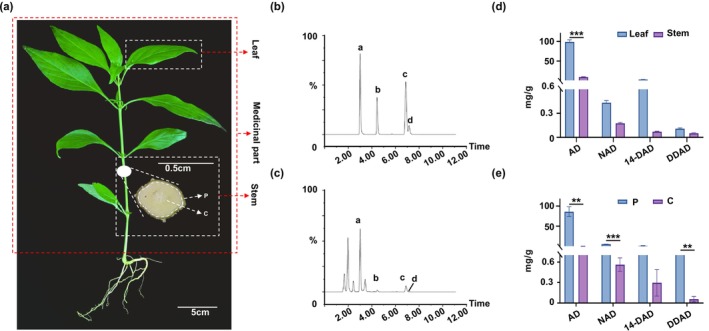
Determination of the content of andrographolide compounds in *A. paniculata* . (a) Samples of *A. paniculata* and different tissues. (b) Chromatogram of the mixed standard. (c) Chromatogram of the *A. paniculata* leaf sample. (d) Determination of the andrographolide compound contents in the leaf and stem. (e) Determination of the andrographolide compound contents in the outer (p) and inner (c) stem tissues. In (d, e), values are shown as means ± standard deviation (*n* = 3 biological replicates). The statistical significance of differences was assessed using a two‐way ANOVA: *p < 0.05, **p < 0.01, ***p < 0.001. The above experiments were performed more than three times, with consistent results obtained across all biological replicates.

This study established a quantitative analytical method for andrographolide‐type diterpenoids (AD: andrographolide; DDAD: dehydroandrographolide; 14‐DAD: 14‐deoxyandrographolide; NAD: neoandrographolide) in different *A. paniculata* tissue using (Figure 1a) LC‐QQQ‐MS/MS. Method validation demonstrated limits of detection (LOD) < 0.10 ng/mL and limits of quantification (LOQ) < 1.00 ng/mL for all analytes (Figure S1), with calibration curves exhibiting R^2^ > 0.99. Chromatographic parameters including molecular formulas, retention times, ion transitions, and linear ranges are detailed in Table S1, confirming that the method is accurate and reliable. Chromatographic alignment confirmed compound identity through retention time concordance between samples and reference standards (Figure 1b,c). Figure 1 presents the quantitative determination results of andrographolide analogs in the leaves and stems of *A. paniculata* . The red dashed box indicates the official medicinal part of the plant (i.e., the entire aerial portion used in medicine), while the white dashed boxes denote the sampling sites in this experiment. Specifically, in the stem transverse section, the area outside the white dashed box represents the peripheral region (designated as P), and the area inside denotes the central region (designated as C) (Figure 1a). Content determination analysis (Figure 1d,e) revealed significantly higher total andrographolide‐type diterpenoids content in leaves (102.12 mg/g fresh weight) versus stems (9.98 mg/g), with andrographolide and 14‐deoxyandrographolide predominating in leaves (Figure 1d) versus andrographolide and neoandrographolide in stems (Figure 1e). Notably, stem peripheral regions showed 185‐fold greater accumulation of andrographolide‐type diterpenoids (55.67 mg/g) than core tissues (0.30 mg/g) (Figure 1e). In summary, the method we established is suitable for the quantification of andrographolide‐type diterpenoids. The content of these compounds was higher in the leaves than in the stems, and within the stems, higher levels were detected in the epidermal layer compared to the inner tissues.

### 2.2 The Optimal Experimental Conditions of DESI‐MSI in *A. paniculata*

**FIGURE 2:**
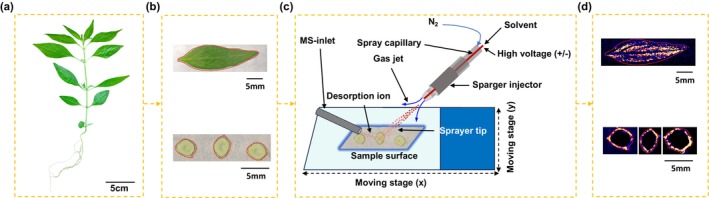
DESI–MSI workflow. (a) Image of the *A. paniculata* plant. (b) Preparation of MSI samples from the leaf and stem tissues. (c) MSI data acquisition. (d) Representative MS images.

**FIGURE 3:**
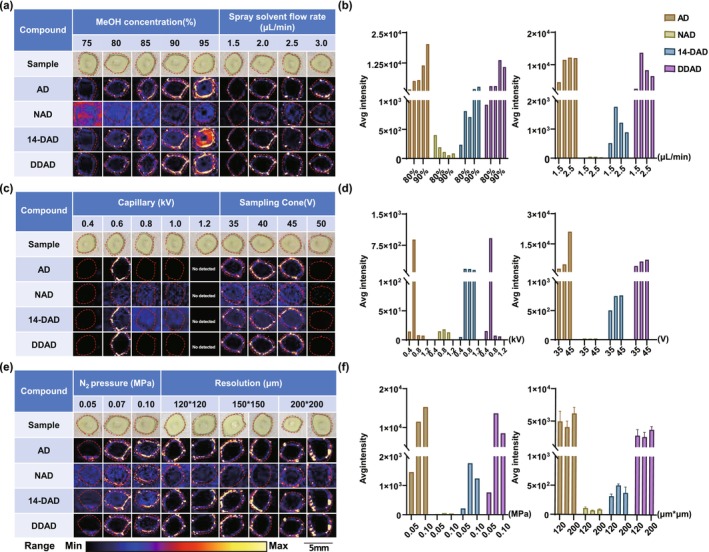
Optimization of the DESI–MSI experimental conditions. (a) MSI of andrographolide components analysed at different MeOH concentrations and spray flow rates. (b) Average intensities of andrographolide components analysed with different MeOH concentrations and spray flow rates. (c) MSI of andrographolide components analysed at different capillary and sampling cone voltages. (d) Average intensities of andrographolide components analysed at different capillary and sampling cone voltages. (e) MSI of andrographolide components analysed with different N_2_ pressures and resolutions. (f) Average intensities of andrographolide components analysed with different N_2_ pressures and resolutions.

Prior to MSI experiments, systematic parameter optimization was performed to establish a high‐resolution spatial metabolomics workflow for andrographolide‐type diterpenoids in *A. paniculata* . The protocol comprises four sequential phases (Figure 2): fresh leaf specimen preparation (Figure 2a), cryosectioning (100 μm thickness, Figure 2b), DESI‐MSI data acquisition (Figure 2c), and pseudocolor visualisation of andrographolide spatial distribution (Figure 2d). A double‐index evaluation system based on target compound MSI effect and average response intensity of MSI was employed (Figure 3). The optimal conditions include the following: (i) mobile phase composition—methanol/water/formic acid (90:10:0.01%, v/v) at 2.0 μL/min flow rate, achieving better ionisation efficiency (Figure 3a,b); (ii) ionisation parameters—0.6 kV capillary voltage and 45 V sampling cone voltage for optimal ion transfer (Figure 3c,d); (iii) nitrogen pressure and spatial resolution: the imaging effect and ionisation efficiency are better when the nitrogen pressure is 0.07 MPa and the pixel size is 150 × 150 μm (Figure 3e,f). These parameters (90% methanol, 2.0 μL/min flow rate, 0.6 kV capillary voltage, 45 V sampling cone voltage, 0.07 MPa nitrogen pressure and 150 × 150 μm pixel size) were ultimately employed to ensure experimental consistency throughout all subsequent procedures.

### 2.3 The Distribution of Andrographolides Mainly Concentrated in Non‐Vein Tissues of Leaves and Outer‐Tissue of Stems

**FIGURE 4:**
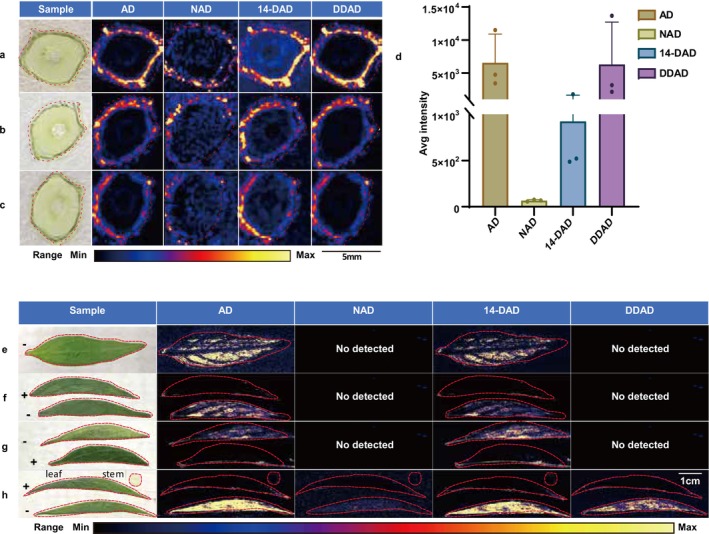
MSI of the leaves and stems of *A. paniculata*. (a–c) MSI of *A. paniculata* stem cross‐sections in three replicate experiments. (d) Average response intensity corresponding to the MSI shown in panels a–c. (e–g) MSI of *A. paniculata* leaf blades in three replicate experiments. (h) Simultaneous MSI of *A. paniculata* leaf blade and stem cross‐section.

Systematic spatial mapping of andrographolide‐type diterpenoids in *A. paniculata* tissues was conducted using optimised MSI parameters (Figure 4). Triplicate stem cross‐sectional analyses (Figure 4a–d) demonstrate clearly defined and spatially congruent signal patterns for AD, DDAD, and 14‐DAD with preferential accumulation in cortical layers. Triplicate leaf surface imaging (Figure 4e–g) reveals that AD and 14‐DAD are specifically enriched in non‐veinal tissue, similar to the chloroplast‐localised biosynthesis hypothesis. Integrated multi‐tissue mapping (Figure 4h) establishes the following metabolite accumulation gradient: leaves > stem periphery. These findings are similar to the result of LC‐QQQ‐MS/MS quantitative analysis, thereby validating the reliability of spatial metabolomics workflows. In conclusion, andrographolide‐type diterpenoids exhibit a tissue‐specific distribution pattern: they are predominantly localised in the non‐vein regions of leaves and are mainly concentrated in the epidermis of stems. While MSI in this study captured only the distribution profiles of andrographolide‐type components within the leaf tissues, precise spatial localization requires further interpretation via scRNA‐seq.

### 2.4 The Specific Expression of ApCPS2 Chiefly Occurred in Mesophyll Cells

**FIGURE 5:**
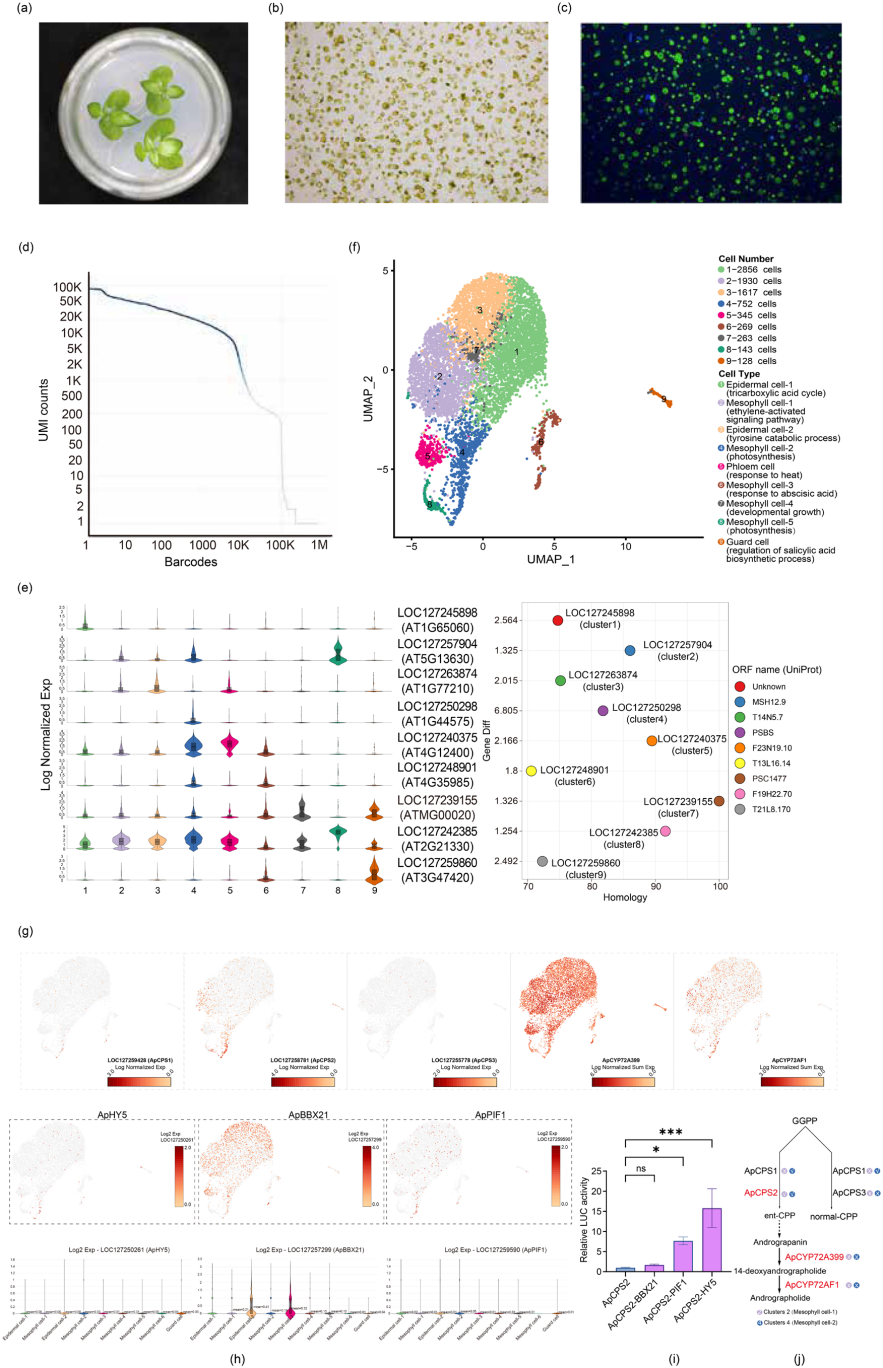
Cell type annotation and gene expression analysis of *A. paniculata* leaves via single cell sequencing. (a, b) Isolation of protoplasts from tissue‐cultured *A. paniculata* leaves. (c) Staining the protoplasts with fluorescein diacetate (FDA). (d) The number of effective cells was quantified using barcodes and UMI counts. (e) Violin plot to show expression levels of *A. paniculata* marker genes with the highest *A. thaliana* homology in each cluster. (f) Visualisation of single‐cell clusters (each distinguished by a unique ID within the same category). (g) Normalised scRNA‐seq expression of *ApCPS1*, *ApCPS2*, *ApCPS3*, *ApCYP72A399* and *ApCYP72AF1*. (h) The expression of *ApHY5*, *ApBBX21*, and *ApPIF1* in different cell groups of *A. paniculata* leaves. (i) Dual‐luciferase reporter assay was used to study the activation of *ApHY5*, *ApBBX21*, and *ApPIF1* with *ApCPS2* promoter in *Nicotiana benthamiana*. Values are shown as means ± standard deviation (*n* = 3 biological replicates). The statistical significance of differences was assessed using a two‐way ANOVA: *p < 0.05, **p < 0.01, ***p < 0.001. The above experiments were performed more than three times, with consistent results obtained across all biological replicates. (j) The andrographolide synthesis pathway involves the participation of *ApCPS2*, *ApCYP72A399*, and *ApCYP72AF1*.

MSI analysis revealed that andrographolide is predominantly localised in the non‐vein tissues of the leaves. To further elucidate its spatial distribution at the cellular level, we subsequently performed scRNA‐seq. A single‐cell suspension was generated via protoplast isolation, yielding approximately 1200 protoplasts/mL from mature leaves (Figure 5a,b). To test the state of the prepared protoplasts, we examined cell viability by staining the protoplasts with fluorescein diacetate (FDA) (Larkin 1976). The survival rate of the protoplasts surpassed 95%. The protoplasts displayed blue‐green fluorescence, and their average maximal PSII quantum yield (Fv/Fm) values exceeded 0.6 as measured by chlorophyll fluorescence. Thus, it was possible to detect andrographolide contents in *A. paniculata* protoplasts using HPLC (Figure S2a–d). These findings suggest that the *A. paniculata* protoplasts possess photosynthetic capability and high viability. Isolated protoplasts were processed using the 10× Genomics Chromium platform, with initial quality control identifying 9421 intact cells. Subsequent filtration excluded doublets, multiplets and apoptotic cells, retaining 8303 cells for downstream analysis (Table S2). Cellular barcoding and UMI quantification revealed a median sequencing depth of 7835 UMIs/cell and detection of 2659 genes/cell (Figure 5d; Table S2, Figure S2e). Unsupervised clustering following dimensionality reduction (t‐SNE) resolved nine transcriptionally distinct cell populations (Figure 5e,f; Table S3), with Cluster 1 representing the predominant subpopulation (2856 cells, 34.4%) and Cluster 9 the rarest (128 cells, 1.6%).

Model plant studies have identified conserved leaf cell markers (Liu et al. 2022). Our de novo *A. paniculata* single‐cell marker identification (Figures S3–S4) and homology‐based alignment of *Arabidopsis* markers (Figure 5e; Table S4) enabled classification based on cell type and gene type (Figure S5). Here, 9 cell type marker genes (with the highest homologous score to *A. thaliana* in each cluster) and corresponding GO:BP (the most significant enrichment results) were used to annotate the *A. paniculata* leaf cell types and biological significances (Tables S4–S5). We ultimately annotated the 9 clusters into four major categories: epidermal cells (ECs), mesophyll cells (MCs), phloem cells, and guard cells (GCs). Clusters 1 and 3 comprised epidermal cells; mesophyll cells populated clusters 2, 4, 6, 7, and 8; phloem‐specific cells were found in cluster 5; and guard cells were enriched in cluster 9 (Figure 5f). In addition, 4‐coumarate: CoA ligase 3 (4CL3, LOC127245898) and SUGAR TRANSPORT PROTEIN 14 (STP14, LOC127263874), expressed specifically in leaf epidermal cells, were enriched in Clusters 1 and 3. PHOTOSYSTEM II SUBUNIT S (PSBS, LOC127250298), a ubiquitous pigment‐binding protein associated with photosystem II (PSII) of land plants, expressed specifically in mesophyll cells, was enriched in Cluster 4. GENOMES UNCOUPLED 5 (GUN5, LOC127257904), MITOCHONDRIAL 26S RIBOSOMAL RNA PROTEIN (RRN26, LOC127239155), and FRUCTOSE‐BISPHOSPHATE ALDOLASE (FBA1, LOC127242385) are expressed specifically in mesophyll cells and enriched in Clusters 2, 7, and 8. In addition, the marker gene LOC127248901, exhibiting the highest homology score in Cluster 6, is annotated as a leaf mesophyll cell marker in *A. thaliana* (homologous to AT4G35985) and encodes the hypothetical protein CDL12_28498 (Figure 5e; Table S4). In summary, single‐cell transcriptomic profiling uncovered extensive cellular diversity within *A. paniculata* leaf tissues.

Key enzymes involved in andrographolide biosynthesis, including *ApCPS1*, *ApCPS2*, *ApCPS3*, *ApCYP72A399*, and *ApCYP72AF1*, were analysed for cell type‐specific expression patterns using single‐cell resolution mapping (Figure 5g; Figure S6). Among these, *ApCPS1* and *ApCPS3* exhibited low expression across all cell clusters, with *ApCPS1* showing restricted expression in mesophyll cell Cluster 4. Although *ApCYP72A399* and *ApCYP72AF1* were ubiquitously expressed at high levels, they lacked cell‐type specificity. Such expression characteristics were often associated with enzymes involved in later steps of specialised metabolic pathways (Wang, Ma, et al. 2025). Notably, *ApCPS2* displayed preferential expression in mesophyll cell Clusters 4, 8, and 2, with the highest specificity observed in Cluster 4 (Figure 5g). This suggested that precursor supply (e.g., ent‐copalyl diphosphate synthesis) may have represented a major regulatory bottleneck in the pathway. Previous studies had also demonstrated that silencing *ApCPS2* markedly reduced andrographolide production and impaired herbivore defence, whereas silencing other *ApCPS* homologues (e.g., *ApCPS1*, *ApCPS3*) had minimal effects (Garg et al. 2024). Regarding the regulatory mechanism, He et al. reported in *Artemisia annua* that light‐responsive transcription factors *AaHY5* and *AaBBX21* upregulate artemisinin biosynthetic gene expression by binding to B‐box motifs in their promoter regions (He et al. 2024). A conserved regulatory mechanism may exist in *A. paniculata* . To investigate this hypothesis, we performed cell type‐specific expression analysis of *ApHY5*, *ApBBX21*, and *ApPIF1*, along with dual‐luciferase reporter assays. Single‐cell data indicated that *ApHY5* and *ApPIF1* were specifically highly expressed in mesophyll cluster 4, exhibiting a spatial expression pattern highly consistent with that of *ApCPS2* (Figure 5h), suggesting their potential synergistic action within the same cell type and formation of a transcriptional regulatory module. Dual‐luciferase assays further confirmed that *ApHY5* and *ApPIF1* can bind to the *ApCPS2* promoter region (Figure S7), positively regulating its transcription and thereby promoting andrographolide biosynthesis and accumulation. As shown in Figure 5i, following their respective interactions with *ApCPS2*, the expression levels of *ApHY5* and *ApPIF1* increased by about 15.80‐fold and 7.69‐fold, respectively, both demonstrating statistically significant differences (*n* = 3). In contrast, although *ApBBX21* was also relatively highly expressed in mesophyll cells (cluster 5), it showed no significant activation effect on *ApCPS2* (Figure 5h,i). In summary, the upstream skeleton synthesis gene *ApCPS2* is specifically expressed in clusters 2 and 4 mesophyll cells, whereas the two downstream modifying P450 genes (*ApCYP72A399* and *ApCYP72AF1*) are ubiquitously expressed across all cell types without significant preference. This expression pattern indicates that mesophyll cells—particularly cluster 4 cells with high *ApCPS2* expression—serve as metabolic hotspots for the biosynthesis of this secondary metabolite, and that the regulatory core of the pathway is likely concentrated at the upstream precursor supply stage. The broad distribution of downstream P450 enzymes may further suggest potential functional redundancy or substrate promiscuity (Figure 5j).

**FIGURE 6:**
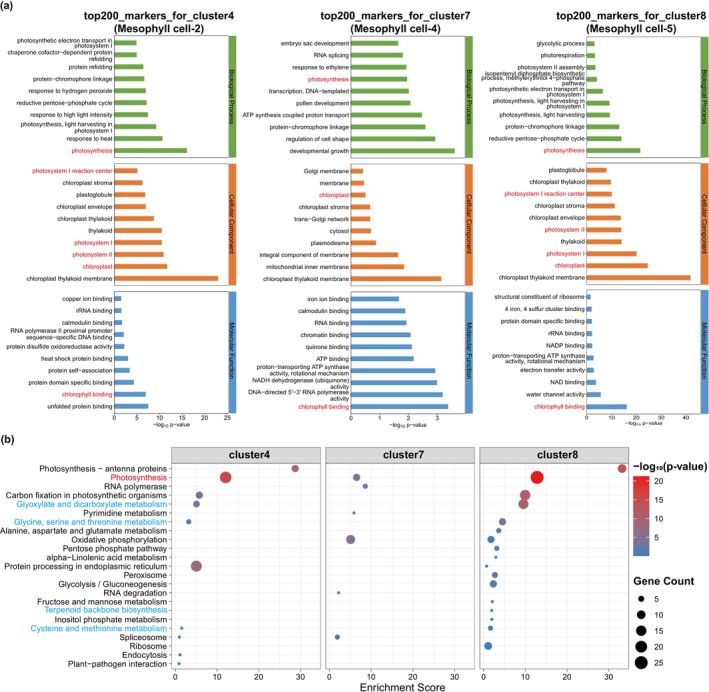
Functional enrichment analysis. (a) GO functional enrichment analysis of clusters 4, 7, 8. (b) KEGG functional enrichment analysis of clusters 4, 7, 8.

To explore the functional relevance of these expression patterns, we performed GO and KEGG enrichment analyses on these clusters (Figure S8). Clusters 4 and 8 showed significant enrichment in photosynthesis‐related biological processes (GO:BP), with corresponding cellular components (GO:CC) and molecular functions (GO:MF) linked to chloroplast thylakoid membranes and chlorophyll binding (Figure 6a; Table S5). Cluster 7, along with Clusters 4 and 8, was identified as mesophyll cells actively engaged in photosynthesis, as further supported by KEGG pathway enrichment (Figure 6b; Table S6). In addition to photosynthesis, Cluster 8 was uniquely enriched in terpenoid backbone biosynthesis, while Clusters 4 and 8 were associated with glycine/serine/threonine metabolism, glyoxylate/dicarboxylate metabolism, and cysteine and methionine metabolism (Figure 6b; Table S6). These pathways collectively regulate terpenoid production (e.g., andrographolide) in *A. paniculata* by modulating carbon flux, energy homeostasis, and sulfur assimilation. Interestingly, the co‐enrichment of photosynthesis and terpenoid‐related pathways in mesophyll cells suggests potential metabolic crosstalk potentially mediated by shared resources such as NADPH (from PPP) and ATP (from oxidative phosphorylation), which are critical for both processes (Vranová et al. 2013). In short, mesophyll cells (clusters 4/7/8) coordinately regulate andrographolide biosynthesis through synergistic interaction between photosynthetic and terpenoid biosynthesis pathways, forming an integrated metabolic network: cluster 8 specifically contributes to terpenoid backbone construction, while clusters 4/8 collectively modulate amino acid metabolism and sulfur assimilation, with their co‐enrichment patterns revealing a NADPH/ATP sharing‐mediated metabolic crosstalk mechanism.

In addition to mesophyll cells, we also conducted GO and KEGG enrichment analyses on other identified cell types within the leaf (Figure S8). Clusters 1 and 3 were identified as epidermal cells. GO biological process (GO:BP) enrichment analysis revealed their predominant association with the tricarboxylic acid cycle, response to insect herbivory, tyrosine catabolic process, and response to sucrose. These findings were corroborated by KEGG pathway analysis, which showed significant enrichment in the citrate cycle (TCA cycle), phenylalanine/tyrosine/tryptophan biosynthesis, tyrosine metabolism, and phenylalanine metabolism. Cluster 5, annotated as vascular bundle cells, exhibited GO‐BP terms including response to heat and high light intensity, alongside KEGG pathways such as thiamine metabolism and glucosinolate biosynthesis. This pattern suggests that light‐ and heat‐responsive biological processes may provide energy and precursors for the associated metabolic activities. Cluster 9, identified as guard cells, was enriched in GO‐BP terms related to regulation of salicylic acid biosynthesis and stomatal opening, as well as KEGG pathways including galactose metabolism and glycerophospholipid metabolism, reflecting their specialised roles in signal transduction and stress adaptation. In summary, epidermal cells primarily function in energy metabolism and defence responses, where enrichment in pathways such as the tricarboxylic acid (TCA) cycle and tyrosine/phenylalanine metabolism provides the energetic and material basis for countering stresses like insect herbivory. Vascular bundle cells exhibit pronounced responsiveness to light and thermal stress, with enriched pathways including thiamine metabolism, indicating their role in converting environmental signals into specific metabolic activities to supply energy and precursors for biosynthesis. Conversely, guard cells are functionally specialised in regulating stomatal behaviour and stress signalling, coordinating processes such as salicylic acid biosynthesis, galactose metabolism, and glycerophospholipid metabolism to modulate stomatal movement and facilitate adaptation to environmental challenges.

All in all, single‐cell RNA sequencing analysis indicated that *ApCPS2* is specifically highly expressed in mesophyll cells. Combined with GO and KEGG enrichment analyses, which confirmed the association of mesophyll cells with photosynthesis and diterpenoid backbone biosynthesis, it is suggested that mesophyll cells may serve as the primary site for andrographolide synthesis.

### 2.5 The Synthesis of Andrographolide Is Influenced by Light

**FIGURE 7:**
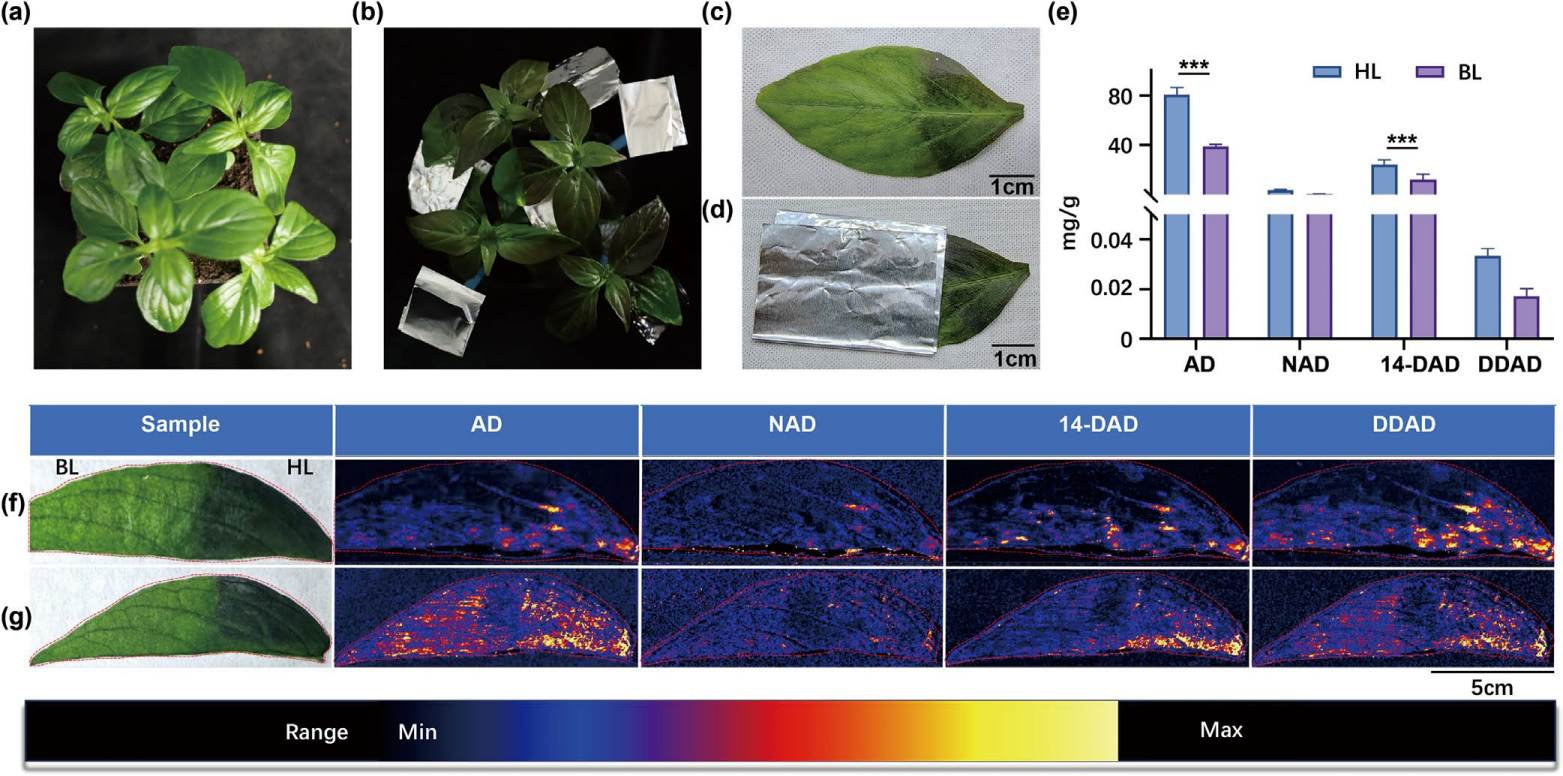
Changes in the phenotypes and contents of *A. paniculata* leaves after light and dark treatments. (a) Experimental materials (31‐day‐old plants). (b) Gross morphology after 7 days of treatment. (c) Comparison of the phenotypes between treatments. (d) Schematic diagram of leaf shading via wrapping in aluminium foil. (e) LCQQQ‐MS/MS quantitative determination of andrographolide compound contents. Values are shown as means ± standard deviation (*n* = 3 biological replicates). The statistical significance of differences was assessed using a two‐way ANOVA: *p < 0.05, **p < 0.01, ***p < 0.001. The above experiments were performed more than three times, with consistent results obtained across all biological replicates. (f, g) Spatial distribution profiles in the adaxial (f) and abaxial (g) leaf surfaces visualised via DESI–MSI.

Figure 7 delineates light‐regulated phenotypes and secondary metabolite accumulation in *A. paniculata* leaves. Experimental materials: Cultivated plants were selected at the third true leaf expansion stage (30‐day growth) (Figure 7a). Light treatment design: distinct regions of the same leaf were partitioned into light and dark groups using physical shading devices (Figure 7b–d). After treatment for 7 days, light‐exposed leaves developed dark‐brown phenotypes (non‐foil‐wrapped regions in Figure 7d), whereas dark controls retained green as before (left panel in Figure 7c). Photochemical dynamics: Maximum photochemical quantum yield (Fv/Fm) was reduced to a relatively low level within 0–24 h under light, then gradually recovering to near‐normal levels by 24–168 h, indicating photo‐damage repair activation (Figure 7c; Figure S9a,b). Quantitative analysis (Figure 7e): LC‐QQQ‐MS/MS revealed about 2.1‐, 4.0‐, 2.3‐, and 1.5‐fold increases in andrographolide, neoandrographolide, 14‐deoxyandrographolide, and dehydroandrographolide levels, respectively, under light (total 2.11‐fold increase vs. dark controls), thus suggesting that optimal light intensity can promote the synthesis of andrographolide components. Spatial metabolomic validation by DESI‐MSI (Figure 7f,g) demonstrated stronger lactone signals in the light group compared to the dark group.

In summary, these findings collectively suggest light intensity‐driven spatial‐specific accumulation of andrographolides in mesophyll tissues, providing mechanistic insights into photoregulation of medicinal compound biosynthesis.

## 3 Discussion

This study establishes an integrated framework combining MSI and scRNA‐seq to elucidate the spatial distribution and biosynthetic regulation of andrographolide‐type diterpenoids in *A. paniculata* . This integrated framework first employed MSI to reveal the spatial distribution pattern of andrographolide‐type components in non‐vein leaf regions, followed by scRNA‐seq for cell type identification and functional validation. The two experimental approaches were sequentially and independently conducted prior to data integration analysis (including non‐vein leaf regions and the cellular distribution of ApCPS2, among others), rather than constituting a co‐localization analysis from a single experimental acquisition. The qualitative overlay analysis of both datasets is visually presented in Figure 5j and detailed in Table S7. Our results demonstrate that andrographolides are preferentially accumulated in non‐veinal leaf tissues and the outer cortex of stems, with mesophyll cells identified as the primary sites for their light‐modulated biosynthesis. Notably, higher levels of andrographolides were detected on the abaxial side of leaves compared to the adaxial side, a difference attributed to leaf surface microstructure influencing detection efficiency—the dense cuticular wax on the adaxial side likely suppresses desorption, whereas the stomata‐rich abaxial side facilitates ionisation. To test this hypothesis, attempts were made to remove the epicuticular wax through cryo‐sectioning and chloroform immersion (Liao et al. 2019) (Figure S10, Supporting Information). However, these preliminary experiments still could not fully eliminate interference from the cuticular layer, nor did they elucidate the intrinsic mechanism underlying the enhanced signal on the abaxial side. It should be emphasised that the differential compound accumulation between the adaxial and abaxial sides may be regulated by multiple factors, including light conditions, translocation efficiency, and inherent structural differences between the two sides, all of which could interfere with quantitative results. Importantly, in comparative experiments involving shaded versus illuminated sections of the same leaf, changes in lactone content were unequivocally attributed to the light variable, robustly confirming the promotive effect of light on the synthesis of these compounds. In conclusion, the mechanism responsible for the differential compound accumulation between the adaxial and abaxial leaf sides of *A. paniculata* requires further systematic investigation. The spatial distribution patterns were robustly supported by complementary data from DESI–MSI and LC‐QQQ‐MS/MS. Operated in full‐scan mode (m/z 100–1200), DESI–MSI enabled untargeted detection of multiple metabolites. Although the study focused primarily on andrographolide and its analogs, the dataset also contains spatial information of other ionizable compounds. By extracting ion images corresponding to characteristic m/z values of known flavonoids (e.g., *apigenin*, *echioidinin*) and phenolic acids (e.g., *ferulic acid*, *chlorogenic acid*), tissue‐specific accumulation of these metabolites can be directly visualised without method re‐optimization (Figure S11), indicating the general applicability of the established MSI workflow for spatial mapping of other plant metabolites. Furthermore, when combined with the light‐induction experiments conducted in this study, this approach could assess whether flavonoids and phenolic acids exhibit similar light‐responsive behaviour, thereby providing a feasible strategy for investigating coordinated regulation across different metabolite classes.

Integrated analysis of MSI and scRNA‐seq data suggested mesophyll cells as the primary biosynthetic sites for andrographolide. Specifically, mesophyll cell clusters (Clusters 4 and 8) exhibited co‐enrichment of photosynthetic pathways and terpenoid backbone biosynthesis, indicating potential metabolic coupling between primary metabolism and diterpenoid biosynthesis. This coupling may be facilitated by shared precursors and energy carriers (e.g., NADPH and ATP) generated through photosynthesis (Huang et al. 2018; Vranová et al. 2013), revealing the metabolic and energetic basis for efficient andrographolide production at the network level. The expression profile of key biosynthetic genes further supported the functional specialisation of mesophyll cells—*ApCPS2*, a critical gene in the diterpenoid biosynthetic pathway, showed highly specific expression in mesophyll cells and co‐localised with the light‐responsive transcription factor *ApHY5* and *ApPIF1* within the same cell type (Figure 5h,i). This spatial expression pattern underscores the central role of cell type‐specific regulation in metabolite accumulation and provides a molecular explanation for the light‐induced enhancement of andrographolide production: as an environmental cue, light directly activates chloroplast function, supplying essential energy (ATP and NADPH) and precursors required for andrographolide biosynthesis via photosynthesis (Krasutsky et al. 2014; Vranová et al. 2013).

Light was selected as the central experimental variable in this study, as opposed to other environmental factors such as soil type, humidity, or temperature, based on the following scientific rationale: first, light has a direct mechanistic link to andrographolide biosynthesis, which predominantly relies on the methylerythritol phosphate (MEP) pathway localised in chloroplasts (Lichtenthaler 1999; Srivastava and Akhila 2010), with light exerting direct regulatory control over chloroplast function and the expression of key MEP pathway genes (Jiang et al. 2022; Lv et al. 2025); second, another reason is that the andrographolide synthesis pathway and key rate‐limiting enzymes are both enriched in mesophyll cells (mesophyll cells perform photosynthesis and are sensitive to light); third, light conditions offer precise controllability, facilitating well‐defined single‐variable experiments by minimising confounding factors, whereas the influence of other environmental variables on metabolite synthesis is likely indirect or non‐specific, making them more suitable for subsequent exploratory studies. Functional validation through light‐induction experiments further corroborated this regulatory mechanism: appropriate light treatment not only enhanced andrographolide synthesis and accumulation but also reinforced its tissue‐specific distribution pattern in mesophyll tissues. Concurrently, the gradual recovery of the Fv/Fm ratio (maximum photochemical efficiency of photosystem II) following initial light‐induced stress indicated the activation of an adaptive photoprotection mechanism, potentially associated with the synthesis of secondary metabolites such as andrographolide—their accumulation may mitigate photo‐oxidative damage to the photosynthetic apparatus, thereby providing functional evidence for the synergistic interaction between light signalling and metabolic regulation.

Although the DESI–MSI workflow established in this study enables spatial detection of multiple metabolites, there remains potential for improvement in resolution and scalability. Future studies could implement more advanced multimodal imaging techniques, such as STAMP (Pitino et al. 2025), to enhance both spatial resolution and metabolite coverage in spatial metabolomics by integrating multiple detection principles, thereby providing technical support for elucidating finer metabolite distribution patterns. Furthermore, while MSI signals of flavonoids and phenolic acids had been preliminarily detected (Figure S11), their spatial distribution characteristics and previously unreported compounds have not been thoroughly investigated. Subsequent research could leverage existing MSI data combined with chemical separation and structural identification techniques to systematically uncover previously unreported chemical constituents in *A. paniculata* and refine its metabolic profile database. At the same time, it could also identify tissue‐specific regulatory factors, establish a multi‐cell type andrographolide biosynthesis system, and increase the overall accumulation of active ingredients. Additionally, integrated multi‐omics analyses–incorporating transcriptomic, metabolomic, and proteomic data–could reveal coordinated regulatory networks among different metabolite classes, offering a more systematic scientific basis for comprehensively understanding secondary metabolism in *A. paniculata* .

The findings of this study provide targeted strategies for the efficient production and utilisation of andrographolide with significant industrial application potential. Specifically, based on the identification of mesophyll cells as the primary biosynthetic sites, the following metabolic engineering approaches can be designed: first, by utilising the *ApHY5* (a mesophyll‐specific promoter) to drive the expression of *ApCPS2* (the key andrographolide biosynthetic gene) in transgenic plants or heterologous expression systems, we could potentially simulate the ecological context of *A. paniculata* leaves. This approach may allow for targeted and high‐yield andrographolide synthesis with enhanced production efficiency and reduced impurities, though its practical application requires further evaluation. Second, optimising light conditions (e.g., intensity, photoperiod, spectral quality) during cultivation to specifically activate metabolic pathways in mesophyll cells, thereby increasing andrographolide content in medicinal materials. Third, establishing cell type‐specific isolation and culture techniques for mesophyll cells to achieve high‐efficiency in vitro production of andrographolide in bioprocessing. Moreover, the integrated “MSI + scRNA‐seq” analytical framework developed in this study exhibits better generalizability and scalability—it can be applied not only to investigate other bioactive compounds in *A. paniculata* but also extended to other medicinally significant plant species, thereby providing a reference for elucidating the biosynthetic mechanisms of active ingredients in diverse medicinal plants and fostering technological and theoretical advances in the study of plant specialised metabolism. The targeted metabolic engineering and standardised cultivation strategies proposed here also offer transferrable practical solutions for enhancing the utilisation efficiency of bioactive compounds in other medicinal plants, contributing substantially to the modernization and industrialization of traditional Chinese medicine.

## 4 Materials and Methods

### 4.1 Reagents and Materials

MeOH and acetonitrile (liquid chromatography–mass spectrometry (LC–MS) grade) were purchased from Thermo Fisher Scientific (Waltham, MA, USA). Leucine‐encephalin was purchased from Thermo Fisher Scientific (Milford, MA, USA). Formic acid was obtained from Shanghai Acmec Biochemical Technology Co. Ltd. (Shanghai, China). The reference standards (≥ 98%, HPLC grade) andrographolide (PRF24071642), neoandrographolide (PRF22122644), 14‐deoxyandrographolide (PRF22112343), and dehydroandrographolide (PRF22072041) were purchased from Chengdu Biopurify Phytochemicals Ltd. (Biopurify, Chengdu, China). All other reagents were of analytical grade.

The *A. paniculata* plant materials were cultivated in a laboratory insect‐proof net house under controlled conditions. Leaf samples were collected from the second pair of leaves from the apex (subapical node), whereas stem samples were obtained from the proximal stem segments (ground‐adjacent, lignified portions). All biological sampling was conducted in September to ensure standardised developmental stages.

### 4.2 LC‐QQQ‐MS/MS Sample Preparation

Fresh *A. paniculata* leaves were excised with sterile scissors, precisely weighed (0.20 g), and transferred to 2 mL microcentrifuge tubes preloaded with three stainless steel grinding beads (5 mm diameter). The tubes were flash‐frozen in liquid nitrogen for 30 s, followed by cryogenic pulverisation using a high‐throughput tissue homogeniser (60 Hz, 2 min). Subsequently, 1 mL of 40% acetonitrile (v/v) was added to the homogenised samples, which were vortexed and sonicated at 40 kHz for 30 min under ambient conditions. After sonication, the samples were cooled to room temperature and centrifuged at 12000 rpm for 10 min. A 10 μL aliquot of the supernatant was diluted to 1000 μL with 40% acetonitrile and centrifuged again (12 000 rpm, 10 min), followed by dilution of 100 μL of the supernatant to 1000 μL with 40% acetonitrile and a final centrifugation step (12 000 rpm, 10 min). The resulting supernatant was filtered through a 0.22 μm microporous membrane and collected in an HPLC vial for subsequent analysis. Identical protocols were applied for stem, epidermal, and internal stem tissue preparations.

### 4.3 Preparation of DESI‐MSI Experimental Samples

Preparation of *A. paniculata* leaf samples: This study systematically compared three preprocessing methods for *A. paniculata* leaf samples. (1) Direct analysis method: fresh leaves were cleaned, dried, and immediately affixed to glass slides using double‐sided tape for direct DESI imaging. (2) PTFE imprinting method: leaves were sandwiched between porous polytetrafluoroethylene (PTFE) membranes and absorbent paper, and metabolites were transferred using a hydraulic press (pressure: 2.0 MPa, time: 25 s). The PTFE membranes were subsequently fixed onto glass slides. (3) Direct compression method: clean, dry leaves were compressed between absorbent papers under identical conditions to moderately disrupt surface structures, followed by fixation of the leaf‐paper composite onto glass slides. Each method was optimised to preserve metabolic integrity while achieving effective sample preparation for high‐throughput analytical measurements.

Preparation of *A. paniculata* stem samples: Fresh stem segments (1.0 cm in length) were collected, rinsed with deionised water, and blotted dry to remove surface moisture. The samples were then embedded and fixed in 5% (w/v) carboxymethyl cellulose sodium (CMC‐Na) embedding medium. Transverse 100 μm thick sections were prepared using a Leica CM1950 cryostat at −20°C. The sections were sequentially adhered to sterile absorbent paper and glass slides via double‐sided tape, followed by storage in a desiccator in the dark for subsequent analysis.

### 4.4 Optimization of the MSI Experimental Conditions

The DESI–MSI parameters were optimised using *A. paniculata* stem cross‐sections under fixed baseline experimental conditions. Spray solvent selection: Five MeOH–formic acid systems (75%–95% MeOH, each containing 0.01% formic acid) were comparatively evaluated based on the imaging quality of andrographolide derivatives. Optimization of the other parameters: A one factor at a time approach was employed to systematically investigate critical instrument settings, including the cone voltage (35–50 V), capillary voltage (0.4–1.2 kV), solvent flow rate (1.5–3.0 μL/min), nitrogen gas pressure (0.05–0.10 MPa), and spatial resolution (120–200 μm). The optimal conditions were determined through iterative testing, where the best combination of parameters was selected on the basis of the highest signal intensity and separation of the target analytes.

### 4.5 LC‐QQQ‐MS/MS and DESI–MSI Analyses

Analysis was performed using a Waters ACQUITY UPLC HSS T3 column (1.8 μm, 2.1 mm × 100.0 mm; Waters Corporation) coupled to a LC‐QQQ‐MS/MS. The optimal ultra‐performance liquid chromatography (UPLC) conditions were as follows: column temperature, ambient temperature; sample compartment temperature, 4°C; flow rate, 0.4 mL/min; and injection volume, 1.0 μL. The mobile phase consisted of water (A) and acetonitrile (B), and gradient elution was performed as follows: 35% B at 0 min, a linear increase to 40% B from 0 to 1 min, maintained at 40% B for 1–10 min, a linear increase to 90% B from 10 to 12 min, a decrease to 35% B from 12 to 13 min, and finally maintained at 35% B until 20 min.

Quantitative analysis was conducted using an UHPLC–QQQ–MS system (UPLC‐Xevo TQ‐Abs MS, Waters Corporation). Nitrogen was employed as the desolvation gas, whereas argon served as the collision gas. The mass spectrometry conditions were as follows: full‐scan mass range, 50–1200 m/z; ion source temperature, 400°C; capillary voltage, 0.6 kV (negative ion mode); and cone voltage, 45 V. Collision energy was applied via a gradient: 3 eV for the low‐energy segment with a linear increase from 25 to 50 eV for the high‐energy segment. Data acquisition was controlled by MassLynx 4.2 software, with real‐time mass correction performed using leucine‐enkephalin (m/z 554.2620) as the lock mass.

DESI–MSI analysis was performed using a Waters Xevo G2‐XS Q‐TOF high‐resolution mass spectrometry system (Waters Corporation, USA) operated in full‐scan negative ion mode. The key instrument parameters were as follows: ion source temperature, 120°C; spray solvent flow rate, 2 μL/min (MeOH:water:formic acid, 90:10:0.01 v/v/v); nitrogen gas pressure, 0.07 MPa; and sampling cone voltage, 45 V. The spray incidence angle was 60°, with a nozzle to sample surface distance of 2 mm, a nozzle to ion transfer tube distance of 6 mm, and an ion transfer tube to sample surface distance of 0.5 mm. The electrospray tip protrusion length was 0.5 mm. Data acquisition was conducted in the range of 50–1200 m/z with a full width at half maximum (FWHM) resolution of 20 000 and a single‐pixel scan time of 0.386 s. Throughout the experiment, leucine‐enkephalin (m/z 554.2620) was employed as a lock mass for real‐time mass axis calibration (Δm < 0.02 Da).

### 4.6 Data Analysis

MSI data processing by computer software and image reconstruction critically and directly impact the analytical outcomes and image quality. Our study employed the following workflow for data analysis: (1) Data preprocessing: the raw data (including mass spectral signals and spatial information) obtained from the DESI–MSI experiments underwent initial processing, including baseline correction, peak detection, and peak intensity quantification, to ensure analytical accuracy. (2) Signal conversion: the mass spectral signals were converted into molecular concentration profiles through normalisation via the use of leucine‐enkephalin (m/z 554.2620) as an internal standard. (3) Image reconstruction: two‐dimensional or three‐dimensional images were generated by mapping molecular spatial distributions onto pixel grids using high‐definition imaging (HDI) software. (4) Image analysis: the edge detection, region segmentation, and feature extraction tools of HDI software were applied to identify target compound localization and distribution patterns.

### 4.7 Preparation of *A. paniculata* Leaf Protoplasts

To isolate protoplasts for scRNA‐seq, the first and second true leaves of 1‐month‐old *A. paniculata* were quickly minced with a blade in a solution of 0.6 M mannitol and then immediately placed in the dark for 30 min. After the sample was filtered through a 40 μm nylon mesh, the tissue fragments were incubated in enzyme solution (1.5% Cellulase R‐10, 0.75% Macerozyme R‐10, 0.6 M mannitol, 20 mM MES at pH 5.7, 20 mM KCl, 10 mM CaCl_2_·2H_2_O, and 0.1% BSA) for 3–4 h in the dark with gentle shaking (60 rpm). Prior to incubation, the enzyme mixture was placed in a 55°C water bath for 10 min to inactivate nonspecific enzymes. After enzymatic digestion, the protoplasts were collected in a round bottom tube by filtering the samples through 40 μm nylon mesh. The protoplasts were collected by horizontal centrifugation at 500 rpm for 5 min. After the supernatant was removed, 5 mL of W5 buffer (2 mM MES at pH 5.7, 154 mM NaCl, 5 mM KCl, and 125 mM CaCl_2_·2H_2_O) was gently added to the tubes. After being washed 2–3 times, the protoplasts were resuspended in WI buffer (200 mM MES, 2 M KCl, and 0.6 M mannitol) before being loaded onto the chromium controller of the 10× Genomics platform. Protoplast viability was determined by trypan blue staining. In the transient gene expression system, the protoplasts were then incubated on ice for 30 min before the supernatant was carefully removed. The protoplasts were subsequently resuspended in MMG buffer (0.6 M mannitol, 15 mM MgCl_2_·6H_2_O, and 4 mM MES at pH 5.7) to a concentration of 1–2 × 10^6^ cells/mL.

### 4.8 scRNA‐Seq Library Construction and Raw Data Processing

Approximately 1 × 10^6^ isolated single cells and enzyme gel beads were packed into a single oil droplet for single‐cell RNA‐seq analysis. The scRNA‐seq library was generated with a single‐cell 3ʹ Library and Gel Bead Kit V3 (10× Genomics, 1 000 075) according to the manufacturer's protocol.

The FASTQ files were processed and aligned to the *A. paniculata* reference genome (RefSeq: GCF_009805555.1; assembly: ASM980555v1) using Cell Ranger software (version 8.0.1) from 10× Genomics, with UMI counts summarised for each barcode. The UMI count matrix was then analysed using the Seurat (version 4.0.0) package in R. To remove low‐quality cells and potential multiplet captures, the following criteria were used: cells were filtered by (1) gene number (< 200), (2) UMI (< 1000), and (3) log10Genes per UMI (< 0.7). The DoubletFinder package (version 2.0.3) was subsequently used to identify potential doublets. To normalise the gene expression data, library size normalisation was performed using the normalizeData function. Specifically, the global scaling normalisation method LogNormalize was applied to normalise gene expression in each cell by total expression, followed by multiplication by a scaling factor (10 000 by default) and log transformation.

The top 2000 highly variable genes (HVGs) were determined using Seurat FindVariableGenes (mean.function = FastExpMean, dispersion.function = FastLogVMR). PCA was performed to reduce the dimensionality with the RunPCA function. The cells were clustered according to their gene expression profile with the FindClusters function and visualised using a 2‐dimensional uniform manifold approximation and projection (UMAP) algorithm with the RunUMAP function. The FindAllMarkers function (test.use = presto) was used to identify the marker genes of each cluster with a log(fold change) threshold of > 0 and minimum percentage of the proportion of gene expression across all cells of > 0.25. GO enrichment and KEGG pathway enrichment analyses of the marker genes (the top 200 genes were selected on the basis of gene differences [gene_diff = pct1/pct2, where pct1 is the proportion of cells expressing the marker gene in the current cluster and pct2 is the proportion of cells expressing the marker gene in other clusters]) were performed using R (version 4.0.3) on the basis of the hypergeometric distribution.

## Author Contributions

H.Z., M.S., and Z.C. contributed equally to this work. H‐.B.W., H‐.L.J., and Y‐.J.C. conceived the idea. H.Z. and M.S. planned and performed experiments. Y.H. provided the materials. H.Z., M.S., Z.C., A.B., M.Z., S.D., Y‐.J.C., and H‐.L.J. contributed to data analysis and interpretation, as well as figure preparation. H.Z., M.S., Z.C., M.Z., Y‐.J.C., and H‐.L.J. wrote and revised the manuscript. H.Z., M.S., X.S., H.Z., Y.H., Y.C., and J.R. contributed to the supplementary experiments. Y‐.J.C., H‐.L.J., and H‐.B.W. supervised the studies.

## Funding

This work was supported by National Natural Science Foundation of China, U22A20446, 32322007, 32100192; Guangdong Major Project of Basic and Applied Basic Research, 2023B0303000022; 2024 Annual Science and Technology Research and Development Incubation Project by the Guangdong Provincial Laboratory of Traditional Chinese Medicine, HQL2024PZ024.

## Conflicts of Interest

The authors declare no conflicts of interest.

## Supporting information


**Figures S1–S11:** pbi70534‐sup‐0001‐FigureS1‐S11.docx.

**Tables S1–S7:** pbi70534‐sup‐0002‐TableS1‐S7.xlsx.

## Data Availability

The raw scRNA‐seq data reported in this paper have been deposited in the Genome Sequence Archive (Genomics, Proteomics & Bioinformatics 2021) of the National Genomics Data Center (Nucleic Acids Res 2022), China National Center for Bioinformation/Beijing Institute of Genomics, Chinese Academy of Sciences (GSA: CRA020160). The data are publicly accessible at https://ngdc.cncb.ac.cn/gsa. The other experimental data that support the findings of this study are available in the Supporting Information of this article.
