# Supplementary material for: Mass Spectrometry Imaging Combined With Single‐Cell Transcriptional Profiling Reveals the Multidimensional Spatial Distributions and Biosynthetic Pathways of Medicinal Components in Andrographis paniculata
Source: Plant Biotechnol J. 2026 Jan 8;24(5):2876–90. doi: 10.1111/pbi.70534 (PMC13110157; doi:10.1111/pbi.70534)
Supplement: Supplementary file 1 — Figures S1–S11: pbi70534‐sup‐0001‐FigureS1‐S11.docx. [file PBI-24-2876-s002.docx]

**Supplementary Figure1**


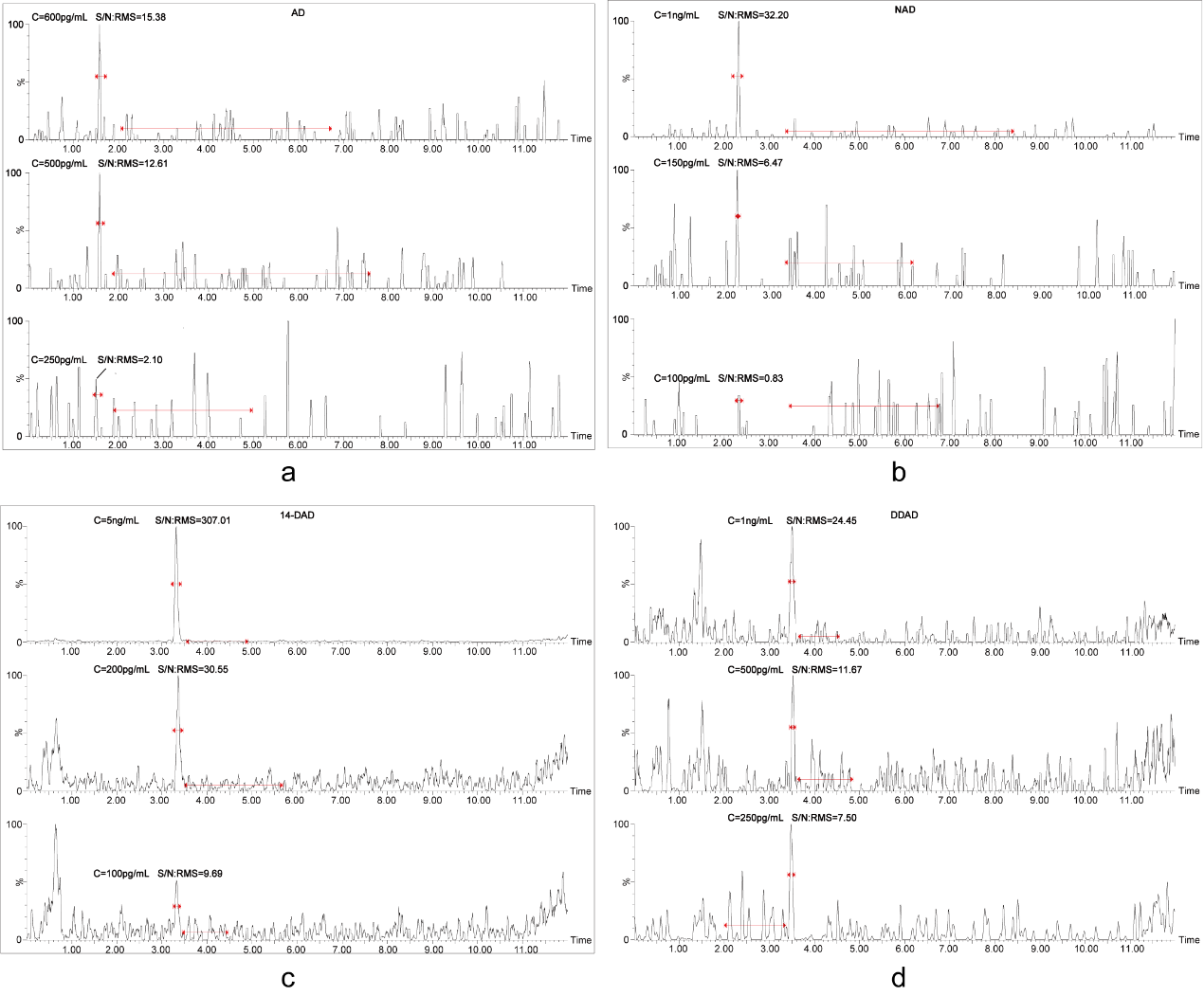


Supplementary Figure 1 presents the analytical validation of limit of quantification (LOQ) and limit of detection (LOD) for andrographolide derivatives: (a) andrographolide, (b) neoandrographolide, (c) deoxyandrographolide, and (d) dehydroandrographolide in *A. paniculata*.

**Supplementary Figure2**


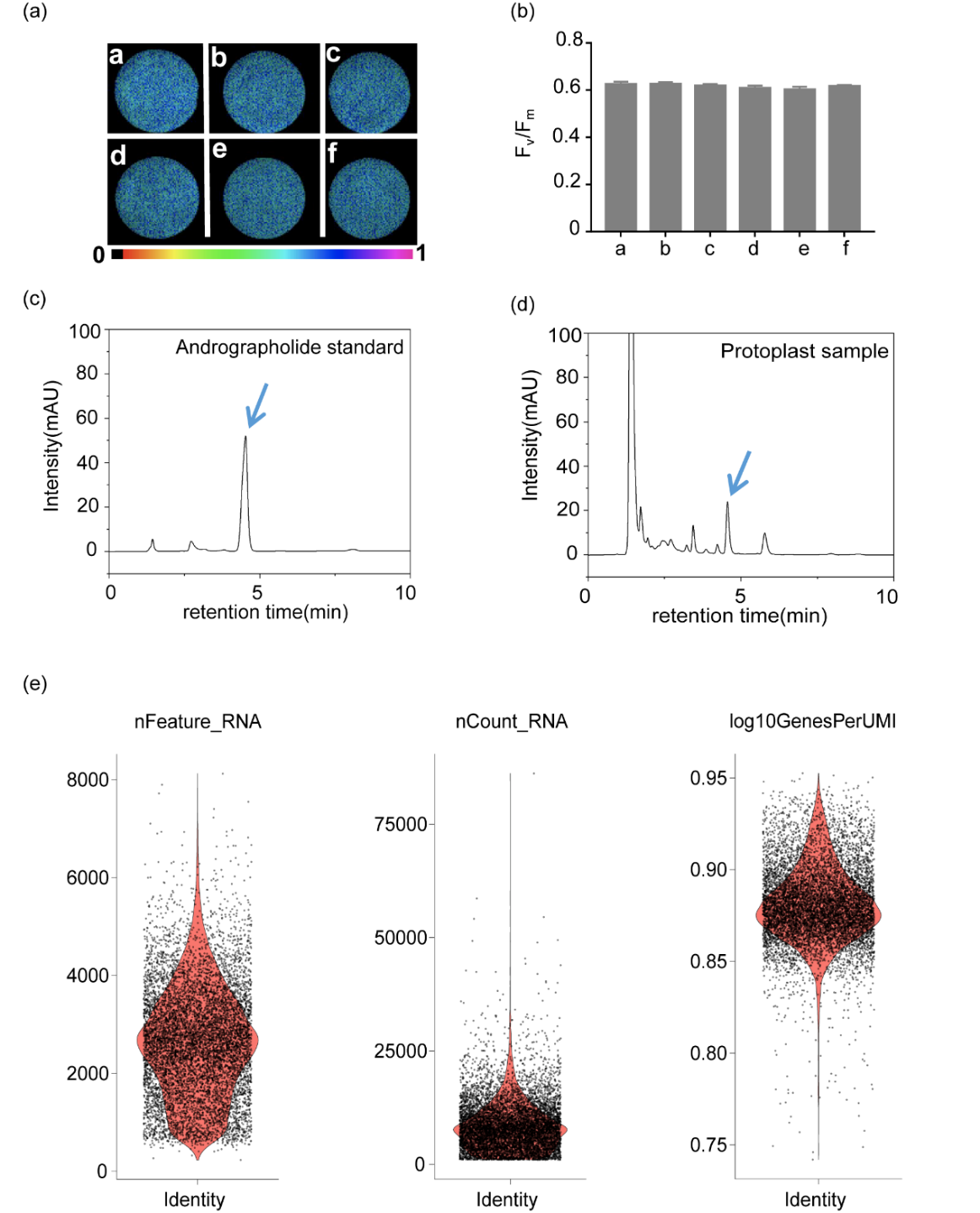


(a) Viability assessment of mesophyll protoplasts in *A. paniculata*; (b) Quantitative analysis of the maximum photochemical quantum yield (Fv/Fm); (c-d) Liquid chromatography profiles of authentic andrographolide standard and protoplast extracts, demonstrating sustained metabolic competence; (e) Violin distribution map of each index content in each cell after quality control: nFeature_RNA and nCount_RNA reflect the number of genes/transcripts expressed in the sample cells, and the cells with abnormal number of genes or transcripts are probably due to the fact that the corresponding water-in-oil droplets contain multiple cells and need to be filtered by setting a reasonable threshold; Log10GenesPerUMI represents the proportion of the number of genes in a unit UMI in the corresponding sample, reflecting the complexity of the data.

**Supplementary Figure3**


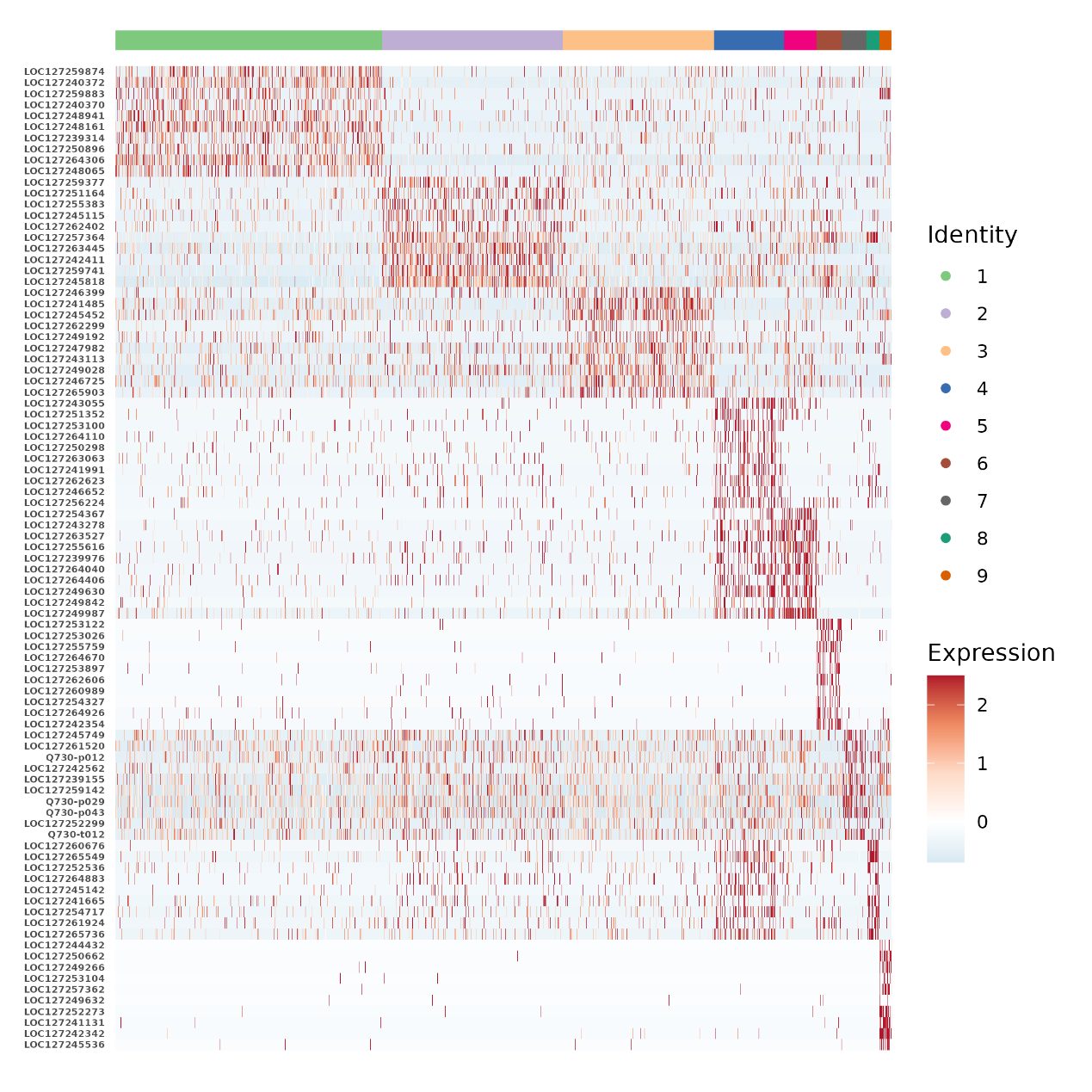


Top10 Marker gene expression heat map: the abscissa is cell group, and the ordinate is Marker gene. In the figure, red indicates high expression and blue indicates low expression.

**Supplementary Figure4**


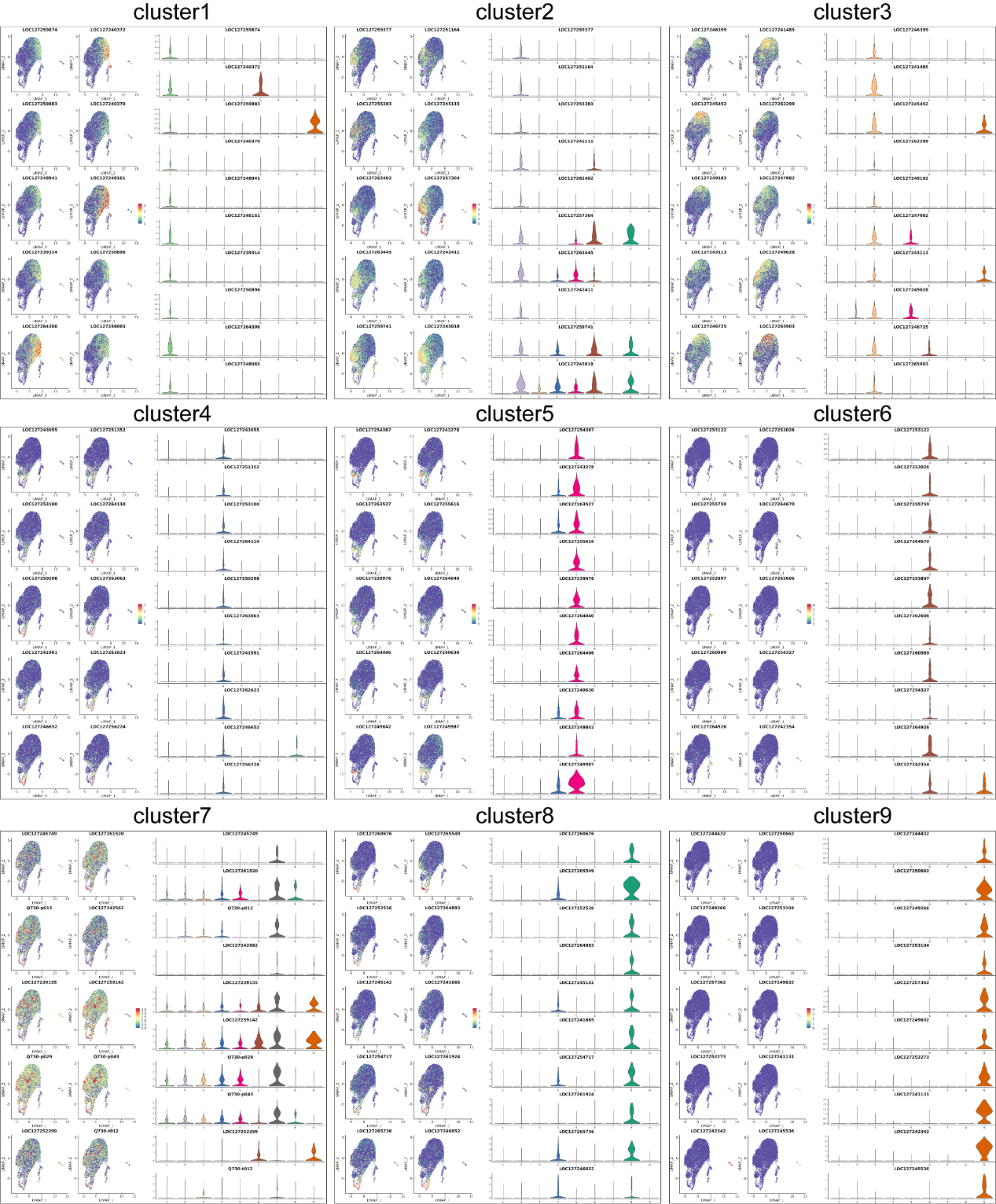


Visualization of Top10 Marker gene in UMAP clustering results: The darker the red color, the higher the expression of the corresponding gene in the cell.

**Supplementary Figure5**


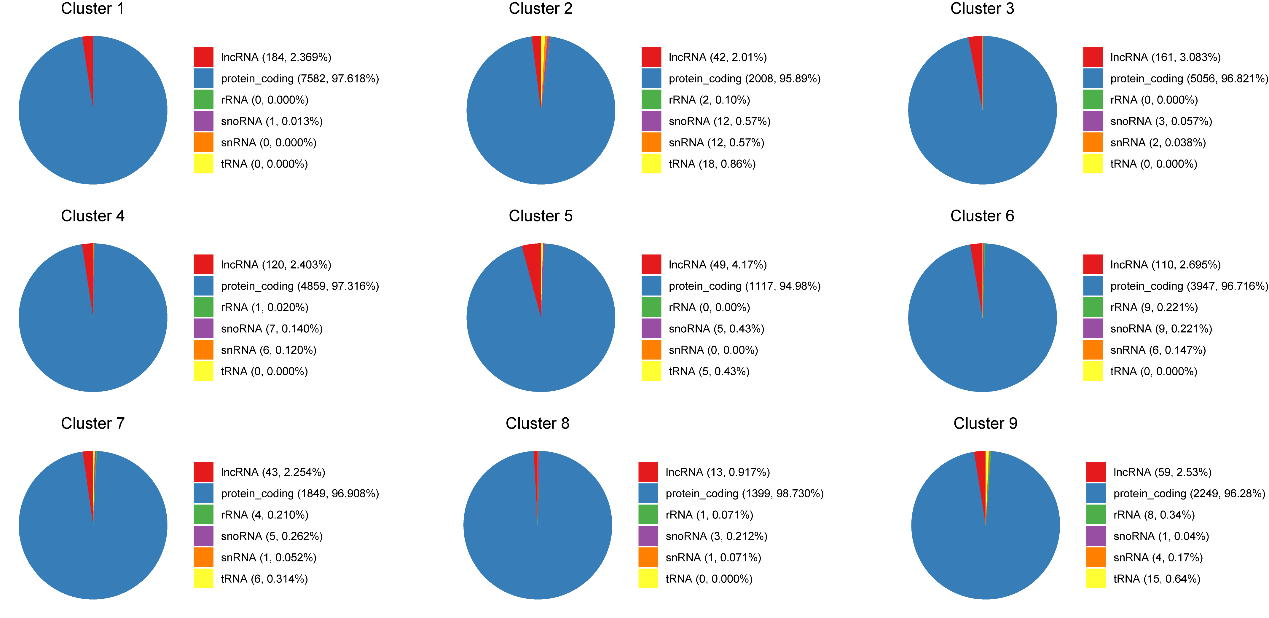


Statistical summary of gene type counts and percentage distribution across single-cell clusters in *A. paniculata*. Long non-coding RNAs (lncRNAs) were prioritized for enumeration, followed by protein-coding genes, with the remaining categories including rRNA, snoRNA, snRNA, and tRNA.

**Supplementary Figure6**


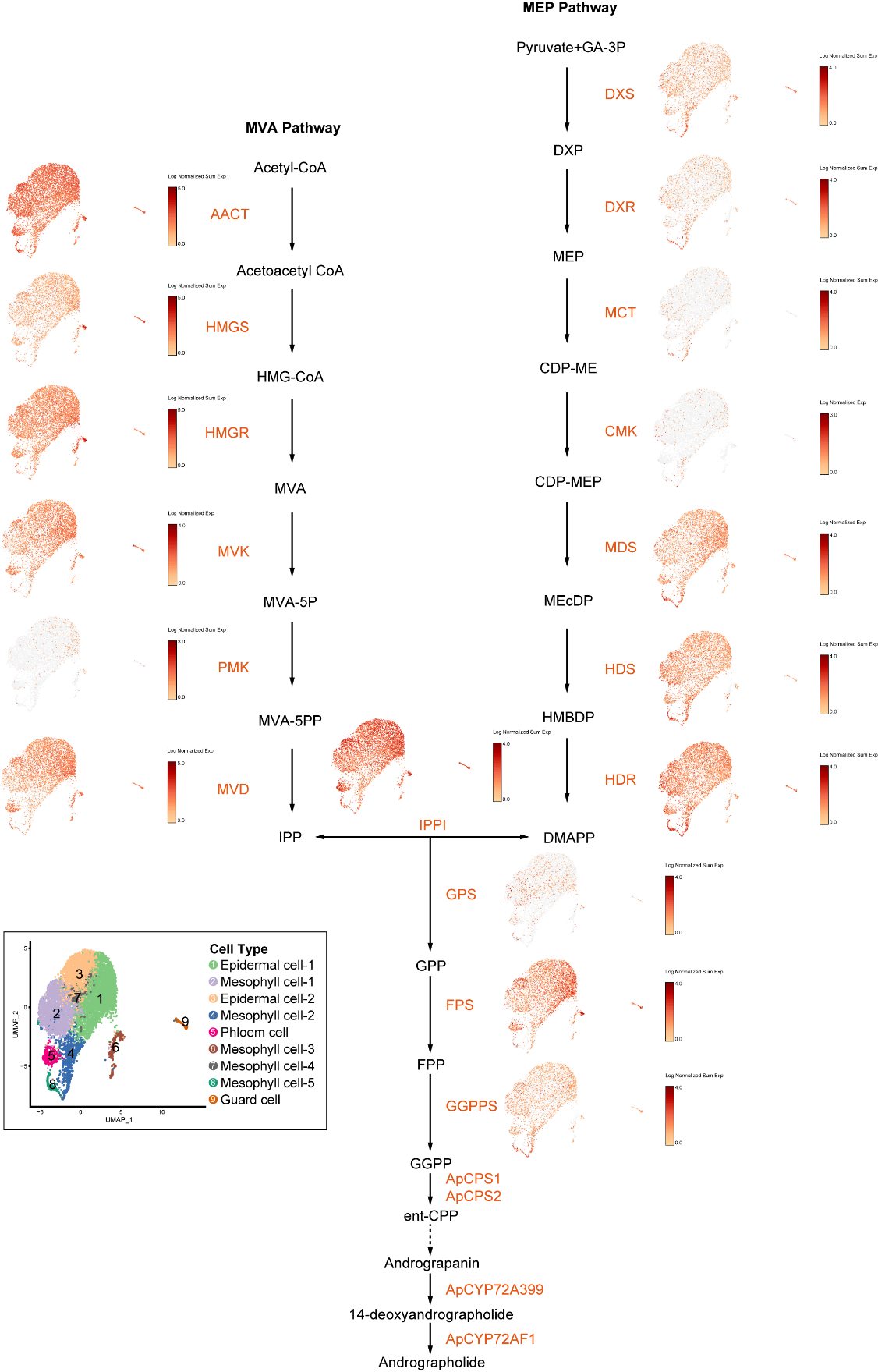


Visualization of the single-cell expression map for key genes in the andrographolide biosynthetic pathway. "Log Normalized Sum Exp" values represent normalized expression levels derived from the summation of all transcript isoforms per gene, while "Log Normalized Exp" indicates normalized expression for genes with a single annotated transcript. The legend displays a diagrammatic sketch of single-cell clusters annotated using *A. thaliana* marker genes.

**Supplementary Figure7**

**
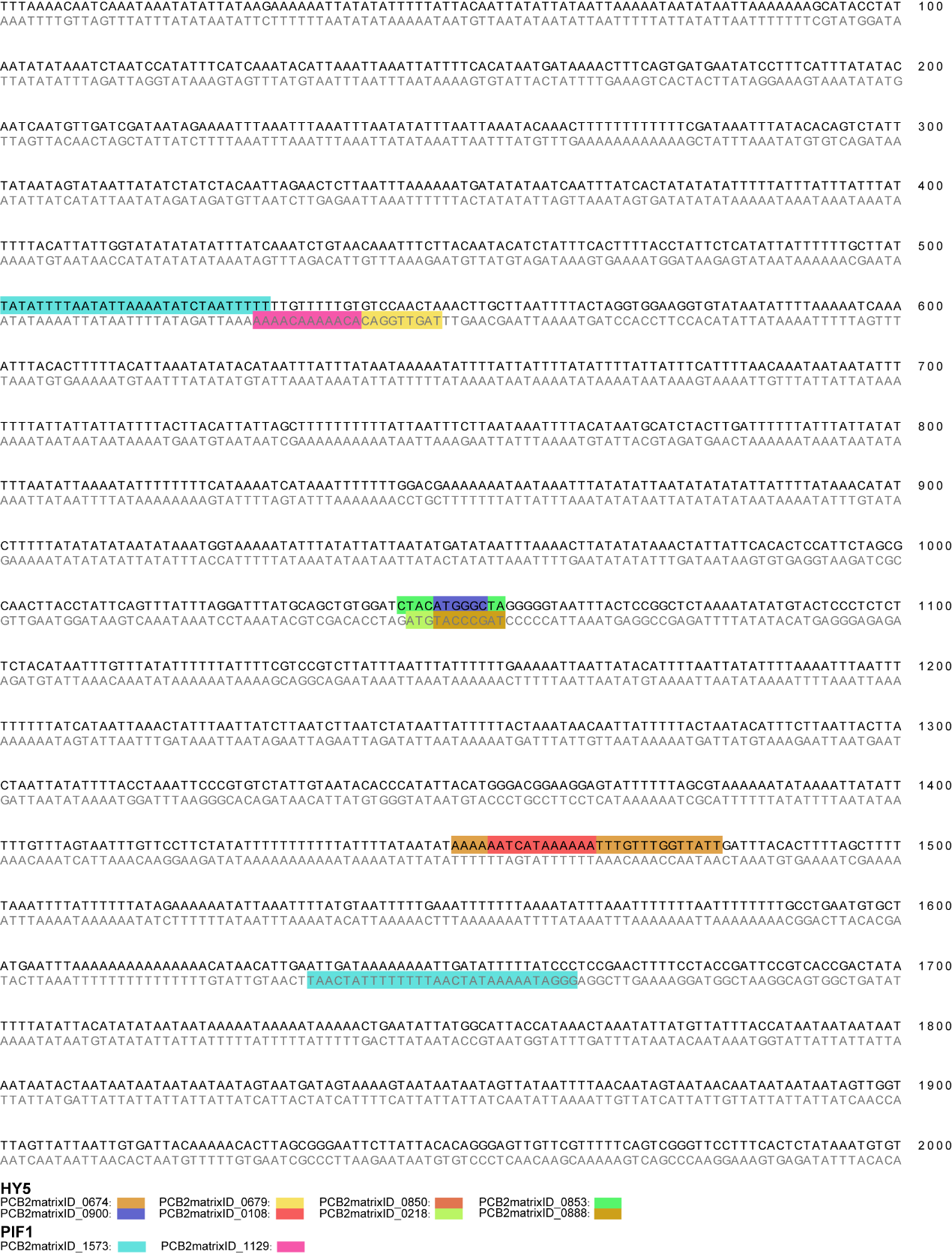
**

Identification of TFBS (Transcription Factor Binding Site, extracted from ChIP-seq data) on *ApCPS2* promoter sequence (generated by PCBase 2.0). The sequences corresponding to TFBS in *ApCPS2* were highlighted using distinct colors.

**Supplementary Figure8

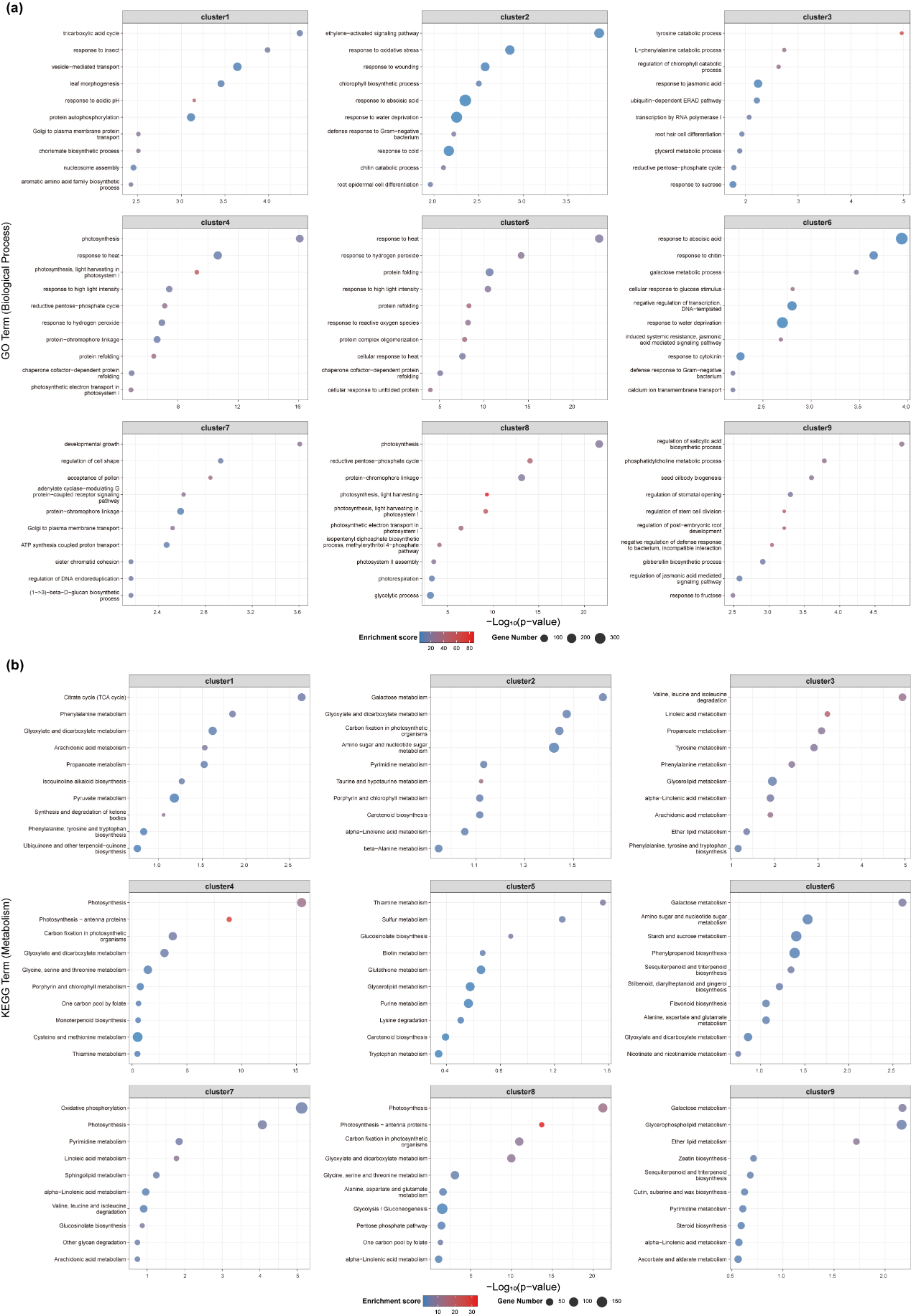
**

The results of GO and KEGG enrichment analysis of 9 clusters identified by *A. paniculata* mesophyll cells, in which (a) is the biological process of GO enrichment to 9 clusters, and (b) is the metabolism of KEGG enrichment to 9 clusters.

**Supplementary Figure9**


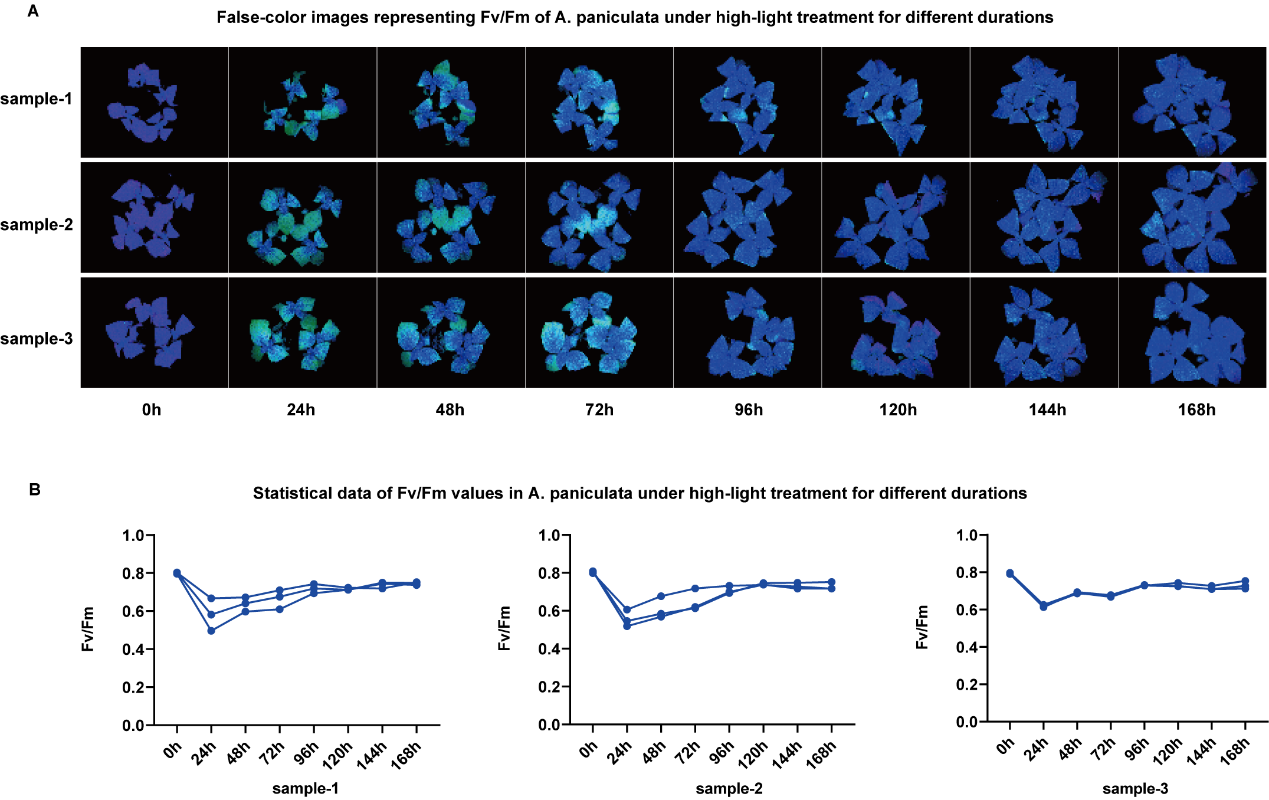


Figure A depicts pseudocolor images of Fv/Fm values in *A. paniculata* under varying high-light durations (Samples 1-3), while Figure B provides corresponding quantitative statistics. Samples 1, 2, and 3 represent three biological replicates.

**Supplementary Figure10**

**
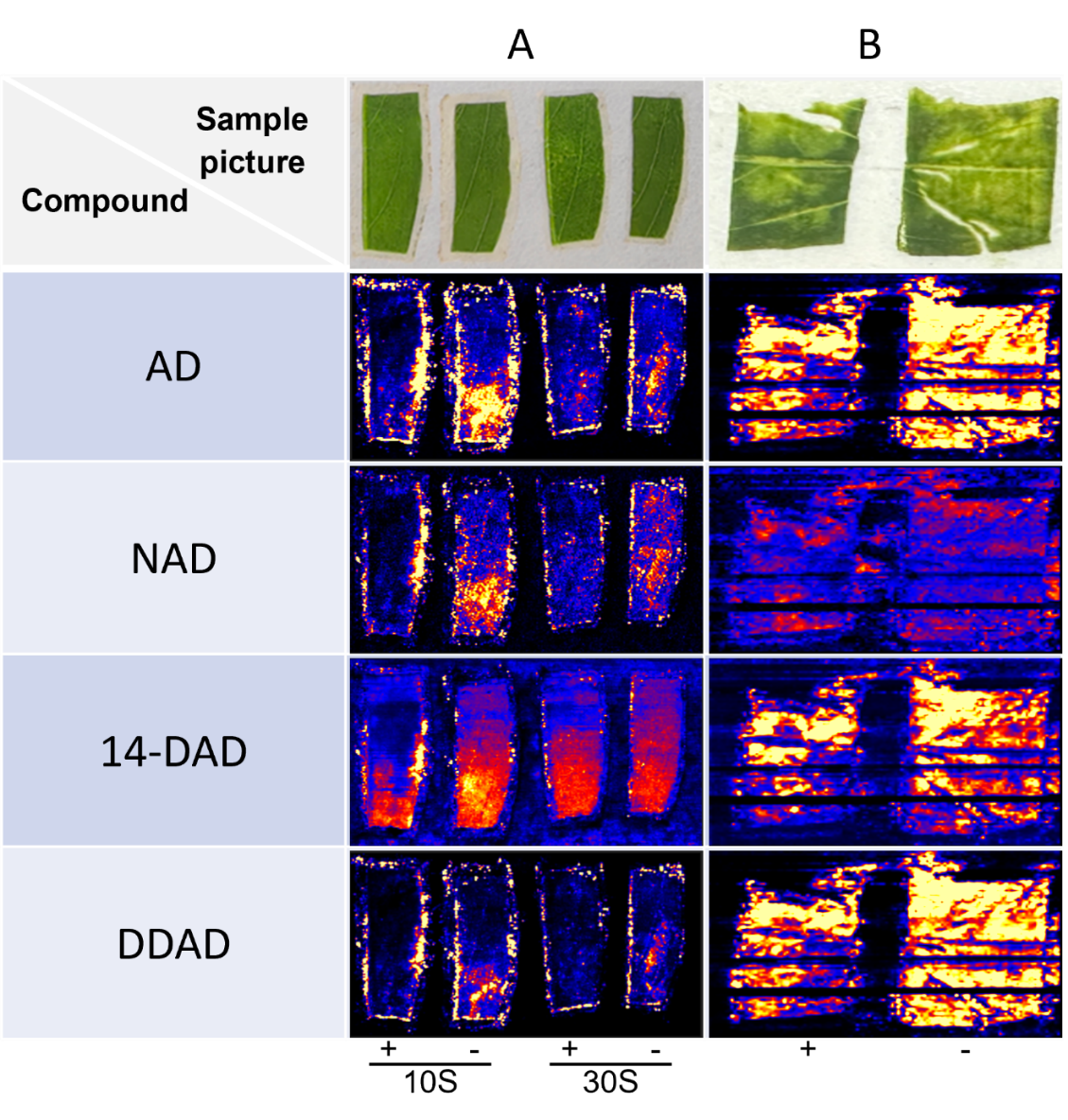
**

Remarks: "+"means the front of *A. paniculata* leaves, "-"means the back of *A. paniculata* leaves, "10S" means chloroform immersion for 10 seconds and "30S" means chloroform immersion for 30 seconds. Preparation of cryosectioned samples: First, the leaf base and apex were excised, and a uniformly sized middle segment was retained and bisected transversely along the direction perpendicular to the midvein. One of the halves was inverted to align the adaxial and abaxial surfaces of the same leaf in the same plane. Subsequently, the sample was affixed onto a porous polytetrafluoroethylene membrane using double-sided adhesive tape, which was then mounted on a glass slide with the sample surface facing upward. Finally, the slide was transferred to a cryostat and sectioned at -20 °C, with both the adaxial and abaxial surface layers simultaneously sectioned to a thickness of approximately 20 μm. The resulting leaf surface was ready for subsequent MSI analysis. Preparation of chloroform-immersed samples: Fresh leaves were immersed in chloroform for 10 seconds, air-dried, and subsequently sandwiched between two layers of blotting paper to form a "blotting paper-leaf-blotting paper" assembly, which was then pressed under 0.6 MPa using a mechanical press. The pressed leaf was adhered to blotting paper with double-sided tape, and the entire composite sample was mounted on a glass slide with the sample surface facing upward for MSI acquisition. The same procedure was applied for the preparation of 30-second chloroform-immersed samples.

A is the results of MSI of the front and back of *A. paniculata* leaves after soaking in chloroform for 10 seconds and 30 seconds respectively; B is the result of MSI after the surface layer of the front and back of *A. paniculata* leaves is removed by cryomicrotome.

**Supplementary Figure11**


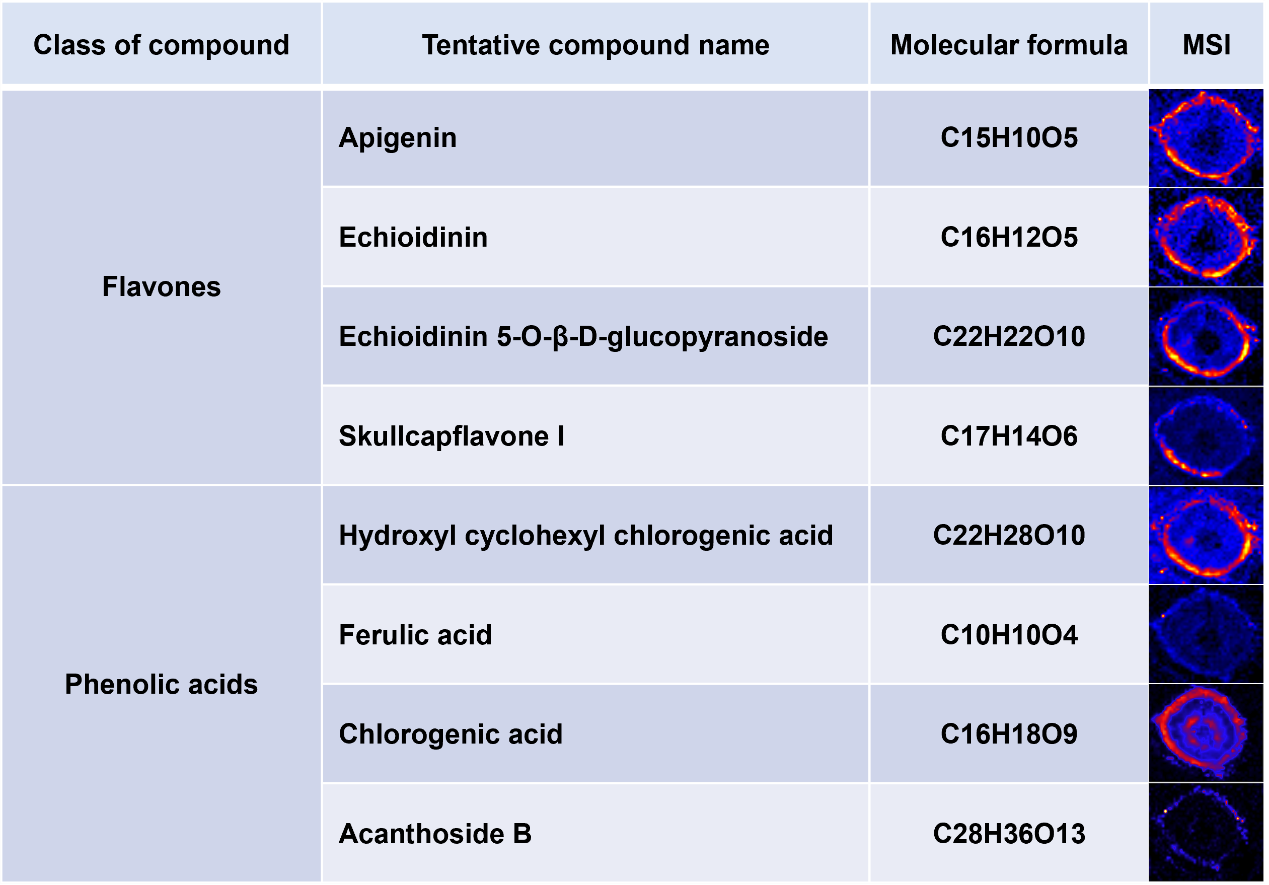


Other flavonoids and phenolic acids with specific spatial distribution observed by MSI.
